# Spatial structure governs the mode of tumour evolution

**DOI:** 10.1038/s41559-021-01615-9

**Published:** 2021-12-23

**Authors:** Robert Noble, Dominik Burri, Cécile Le Sueur, Jeanne Lemant, Yannick Viossat, Jakob Nikolas Kather, Niko Beerenwinkel

**Affiliations:** 1 Department of Biosystems Science and Engineering, ETH Zurich, Basel, Switzerland; 2 SIB Swiss Institute of Bioinformatics, Basel, Switzerland; 3 Department of Evolutionary Biology and Environmental Studies, University of Zurich, Zurich, Switzerland; 4 Biozentrum, University of Basel, Basel, Switzerland; 5 Ceremade, Université Paris Dauphine-PSL, Paris, France; 6 German Cancer Consortium (DKTK), Heidelberg, Germany; 7 Applied Tumor Immunity, German Cancer Research Center (DKFZ), Heidelberg, Germany; 8 Internal Medicine III, University Hospital RWTH Aachen, Aachen, Germany; 9 Present Address: Department of Mathematics, City, University of London, London, UK

**Keywords:** Cancer genetics, Population genetics

## Abstract

Characterizing the mode—the way, manner or pattern—of evolution in tumours is important for clinical forecasting and optimizing cancer treatment. Sequencing studies have inferred various modes, including branching, punctuated and neutral evolution, but it is unclear why a particular pattern predominates in any given tumour. Here we propose that tumour architecture is key to explaining the variety of observed genetic patterns. We examine this hypothesis using spatially explicit population genetics models and demonstrate that, within biologically relevant parameter ranges, different spatial structures can generate four tumour evolutionary modes: rapid clonal expansion, progressive diversification, branching evolution and effectively almost neutral evolution. Quantitative indices for describing and classifying these evolutionary modes are presented. Using these indices, we show that our model predictions are consistent with empirical observations for cancer types with corresponding spatial structures. The manner of cell dispersal and the range of cell–cell interactions are found to be essential factors in accurately characterizing, forecasting and controlling tumour evolution.

## Abstract (web summary)

Tumour evolution modelling indicates that different tumour spatial structures can determine different tumour evolutionary modes, which are regulated by cell dispersal and cell–cell interactions. Model predictions of four evolutionary modes are consistent with empirical observations of cancers with varying architectures.

## Main

A tumour is a product of somatic evolution in which mutation, selection, genetic drift and cell dispersal generate a patchwork of cell subpopulations (clones) with varying degrees of aggressiveness and treatment sensitivity^1^. A primary goal of modern cancer research is to characterize this evolutionary process to enable precise, patient-specific prognoses and optimize targeted therapy regimens. However, studies revealing the evolutionary features of particular cancers raise as many questions as they answer. Why do different tumour types exhibit different modes of evolution^2–8^? What conditions sustain the frequently observed pattern of branching evolution, in which clones diverge and evolve in parallel^2,9–11^? And why do some pan-cancer analyses indicate that many tumours evolve neutrally^12^, whereas others support extensive selection^13^?

**Fig. 1:**
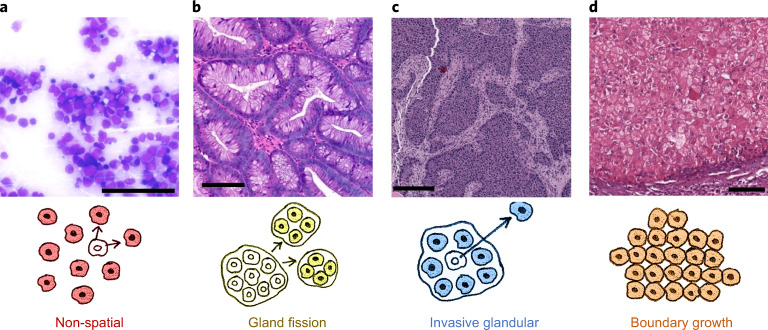
Representative regions of histology slides from human tumours exemplifying four different kinds of tissue structure and manners of cell dispersal. **a**, Acute myeloid leukaemia, M2 subtype, bone marrow smear. **b**, Colorectal adenoma. **c**, Breast cancer (patient TCGA-49-AARR, slide 01Z-00-DX1). **d**, Hepatocellular carcinoma (patient TCGA-CC-5258, slide 01Z-00-DX1). Image **a** is courtesy of Cleo-Aron Weis; image **b** is copyright St Hill et al. (2009)^91^ and is used here under the terms of a Creative Commons Attribution License; images **c** and **d** were retrieved from TCGA at https://portal.gdc.cancer.gov, with brightness and contrast adjusted linearly for better visibility. Scale bars, 100 μm. The illustration below each histology image describes the corresponding types of spatial structure and cell dispersal.

Factors proposed as contributing to tumour evolution include microenvironmental heterogeneity, niche construction and positive ecological interactions between clones^1,14–17^. However, because such factors have not been well characterized across human cancer types, it remains unclear how they might relate to evolutionary modes. In contrast, it is well established that tumours exhibit a wide range of architectures and types of cell dispersal^18,19^ (Fig. 1), the evolutionary effects of which have not been systematically examined. Because gene flow (the transfer of genetic information between localized populations^20^) is a principal force in evolutionary dynamics, we hypothesized that different tumour structures might result in different evolutionary modes. To test this hypothesis, we developed a way to formulate multiple classes of mathematical models, each tailored to a different class of tumour, within a single general framework, and we implemented this framework as a stochastic computer programme.

Our modelling approach is built on basic tenets of cancer evolutionary theory^1^. Simulated tumours arise from a single cell that has acquired a fitness-enhancing mutation. Each time a tumour cell divides, its daughter cells can acquire passenger mutations, which have no fitness effect, and more rarely driver mutations, which confer a fitness advantage. In solid tumours, we assume that cells compete with one another for space and other resources. Whereas previous studies have assumed that tumours grow into empty space, our model also allows us to simulate the invasion of normal tissue—a defining feature of malignancy.

## Results

### Tumour architecture can determine the mode of evolution

To test whether varying tumour architecture suffices to alter the tumour evolutionary mode, we considered four particular models with different spatial structures and manners of cell dispersal but identical evolutionary parameters (driver mutation rate and distribution of driver fitness effects). We set the dispersal probability per cell division such that all tumours take a similar amount of time to grow from one cell to one million cells, corresponding to several years in real time.

**Fig. 2:**
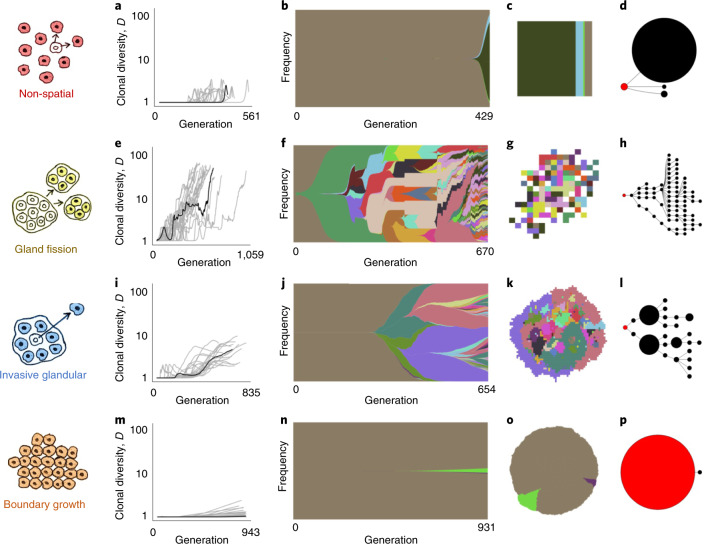
Four modes of tumour evolution predicted by our model. **a**, Dynamics of clonal diversity (inverse Simpson index *D*) in 20 stochastic simulations of a non-spatial model. Black curves correspond to the individual simulations illustrated in subsequent panels (having values of *D* and mean number of driver mutations *n* closest to the medians of sets of 100 replicates). **b**, Muller plot of clonal dynamics over time, for one simulated tumour according to the non-spatial model. Colours represent clones with distinct combinations of driver mutations (the original clone is grey-brown; subsequent clones are coloured using a recycled palette of 26 colours). Descendant clones are shown emerging from inside their parents. **c**, Final clone proportions. **d**, Driver phylogenetic trees. Node size corresponds to clone population size at the final time point and the founding clone is coloured red. Only clones whose descendants represent at least 1% of the final population are shown. **e**–**h**, Results of a model of tumour growth via gland fission (8,192 cells per gland). In the spatial plot (**g**), each pixel corresponds to a patch of cells, corresponding to a tumour gland, coloured according to the most abundant clone within the patch. **i**–**l**, Results of a model in which tumour cells disperse between neighbouring glands and invade normal tissue (512 cells per gland). **m**–**p**, Results of a boundary-growth model of a non-glandular tumour. In all cases, the driver mutation rate is 10^−5^ per cell division, and driver fitness effects are drawn from an exponential distribution with mean 0.1. Other parameter values are listed in Supplementary Table 4.

Our first case is a non-spatial model that has been proposed as appropriate to leukaemia^21,22^, a tumour type in which mutated stem cells in semi-solid bone marrow produce cancer cells that mix and proliferate in the bloodstream (Fig. 1a). When simulating tumour growth in the absence of spatial constraints, rapid clonal expansions can result from driver mutations that increase the cell division rate by as little as a few percent, and the vast majority of cells eventually share the same set of driver mutations (Fig. 2a–d). These characteristics are reminiscent of chronic myeloid leukaemia, in which cell proliferation is driven by a single change to the genome^23^, and acute myeloid leukaemia, which has relatively few drivers^24^.

**Extended Data Fig. 1:**
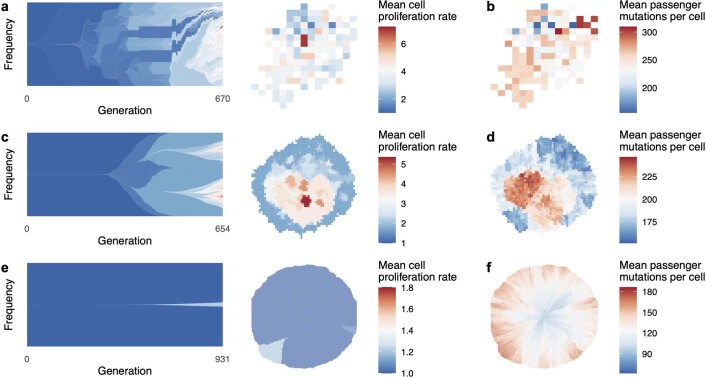
Cell proliferation rates and mutation burdens. a, Evolution and final spatial distribution of cell proliferation rates, for a model of tumour growth via gland fission (8,192 cells per gland). b, Final spatial distribution of mutation burdens. c-d, Results of a model in which tumour cells disperse between neighbouring glands and invade normal tissue (512 cells per gland). e-f, Results of a boundary-growth model of a non-glandular tumour. In all cases, the driver mutation rate is 10^−5^ per cell division, and driver fitness effects are drawn from an exponential distribution with mean 0.1. Other parameter values are listed in Supplementary Table 4.

**Extended Data Fig. 2:**
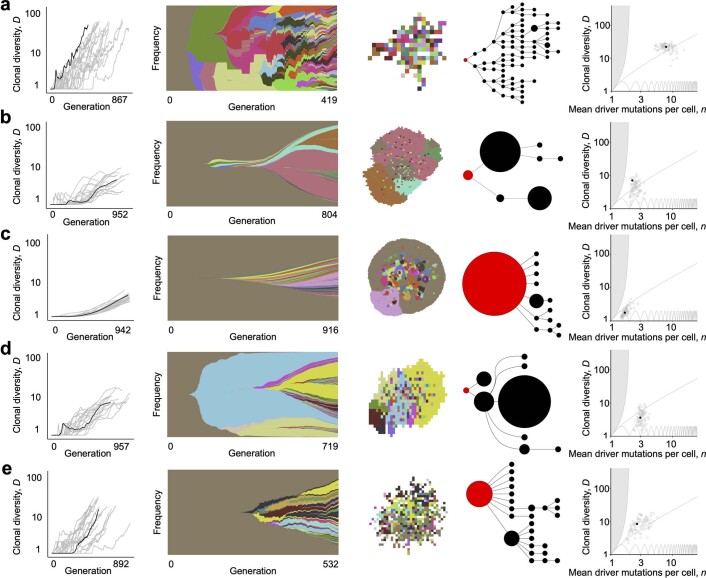
Results of variant models. First column: Dynamics of driver mutation diversity in 20 stochastic simulations. Dynamics of clonal diversity (inverse Simpson index *D*) in 20 stochastic simulations of a non-spatial model. Black curves correspond to the individual simulations illustrated in subsequent columns. These particular simulations are those with indices *D* and *n* closest to the medians of sets of 100 replicates. Second column: Muller plots of clonal dynamics over time. Colours represent clones with distinct combinations of driver mutations (the original clone is grey-brown; subsequent clones are coloured using a recycled palette of 26 colours). Descendant clones are shown emerging from inside their parents. Third column: Final clone proportions (for the non-spatial model) or spatial arrangement (for spatial models). For spatial models, each pixel corresponds to a patch of cells, corresponding to a tumour gland, coloured according to the most abundant clone within the patch. Fourth column: Driver phylogenetic trees. Node size corresponds to clone population size at the final time point and the founding clone is coloured red. Only clones whose descendants represent at least 1% of the final population are shown. Final column: Evolutionary indices *D* and *n* at the final time point. Black points correspond to the individual simulations illustrated in previous columns. a, A model of tumour growth via gland fission (8,192 cells per gland), in which cells can acquire driver mutations that increase their contribution to the gland fission rate (with an average effect size of 50%), in addition to drivers that increase the cell division rate. b, A model in which tumour cells invade normal tissue but do not disperse within the tumour bulk (512 cells per gland). c, A boundary-growth model of a non-glandular tumour in which cells invade neighbouring sites within the tumour. d, A model in which tumour cells invade normal tissue at the tumour boundary only (2,048 cells per gland). e, A model of tumour growth via gland fission (2,048 cells per gland). Other parameter values are listed in Supplementary Table 4.

In our second model, consistent with the biology of colorectal adenoma^25^ and in common with previous computational models of colorectal carcinoma^5,26,27^, we simulate a tumour that consists of large glands (Fig. 1b) and grows via gland fission (bifurcation). Although the driver mutation rate and the fitness effect are exactly the same as in the previous case, the addition of spatial structure dramatically alters the mode of tumour evolution. The organization of cells into glands limits the extent to which driver mutations can spread through the population, so that selective sweeps become progressively localized as the tumour expands. For our parameter values, this process leads to a highly branched, fan-like driver phylogenetic tree and ever greater spatial diversity, with different combinations of driver mutations predominating even in neighbouring glands (Fig. 2e–h). The mean tumour cell fitness increases substantially, but there is also extensive, positively correlated intratumour variation in cell fitness values and passenger mutation counts (Extended Data Fig. 1a–b). Model outcomes are similar even if cells are able to acquire drivers that directly increase the gland fission rate, because such mutations rarely spread within glands (Extended Data Fig. 2a).

**Extended Data Fig. 3:**
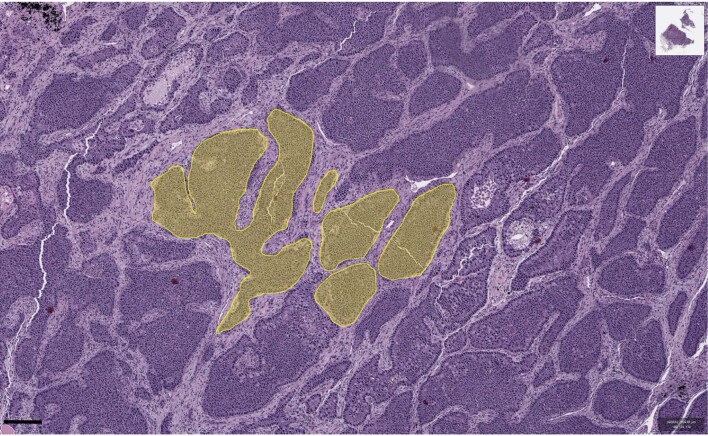
Quantification of tumour cell numbers per gland in a breast cancer histology slide. Multiple glands were manually outlined and the number of cells in each gland was counted automatically. Shaded areas are cell masks obtained with QuPath. The original image was retrieved from The Cancer Genome Atlas at https://portal.gdc.cancer.gov (patient TCGA-49-AARR, slide 01Z-00-DX1).

**Extended Data Fig. 4:**
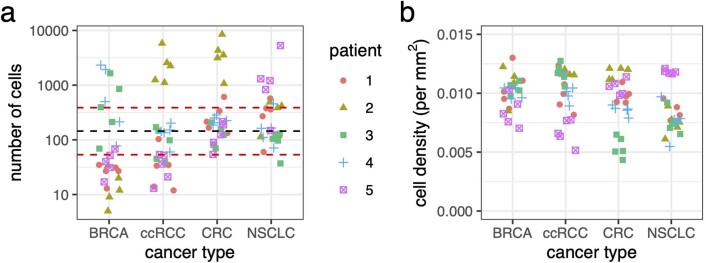
Results of semi-automated analysis of five glands in each of twenty randomly selected TCGA histology slides, representing four cancer types. a, Number of cells per gland (dashed lines correspond to the overall median, 25% and 75% quantiles). b, Cell density. BRCA = breast invasive carcinoma; ccRCC = clear cell renal cell carcinoma; CRC = colorectal cancer; NSCLC = non-small-cell lung cancer.

The third case corresponds to a glandular tumour that grows by invading adjacent normal tissue, as documented in various types of solid tumour, including many colorectal, breast and lung cancers^19,28^. Glandular tumours are subdivided into localized cell communities (Fig. 1c), whose small size has previously been inferred by community detection methods^29^ and mathematical modelling.^30^ To obtain additional estimates of gland size in four cancer types, we used semi-automated analysis of histology slides (Extended Data Fig. 3) and found that each gland contains between a few hundred and a few thousand cells (Extended Data Fig. 4a). In simulations with gland sizes within this range, we find that even small increases in cell fitness can spark rapid clonal expansions. Clonal interference nevertheless inhibits selective sweeps, resulting in a zonal tumour in which large regions share the same combination of driver mutations (Fig. 2i–l and Extended Data Fig. 1c,d). Simulated invasive glandular tumours typically exhibit stepwise increases in driver diversity and a phylogeny with several long branches, qualitatively consistent with observations in numerous cancer types^2,3,11^. Restricting cell dispersal to the tumour boundary without dispersal within the tumour bulk (to simulate tumours that lack intratumoural budding^28^ or tumours in which proliferation is confined to the boundary^31^) results in somewhat shorter branches (Extended Data Fig. 2b).

Our fourth and final model represents a tumour with no glandular structure and with growth confined to its boundary (Fig. 1d). Expansive tumour growth associated with a clearly defined boundary and no sign of active migration occurs in tissues that impose relatively weak physical resistance^18^. Boundary-growth models have in particular been proposed as appropriate for simulating the evolution of certain kinds of hepatocellular carcinoma^7,32^, although it should be noted that hepatocellular carcinoma in general exhibits a wide range of growth patterns^33^. The spatial structure of the boundary-growth model favours genetic drift, rather than selection. For our fixed parameter values, tumour evolution in this case is effectively almost neutral (Fig. 2m–p and Extended Data Fig. 1e), and mutations can spread only by surfing on a wave of population expansion^34–36^. Consequently, the mutation burden generally increases from the tumour core to its boundary (Extended Data Fig. 1f). Selection is only slightly more prominent when cells can compete with their nearest neighbours within the tumour mass (Extended Data Fig. 2c). Suppression of selection in the boundary-growth model is consistent with evidence of effectively neutral evolution in hepatocellular carcinoma^7^, as well as the existence of large, well-differentiated benign tumours such as leiomyomas^37^ and fibroadenomas^38^ that only rarely progress to malignancy.

### Characterization of evolutionary modes and comparison with data

**Fig. 3:**
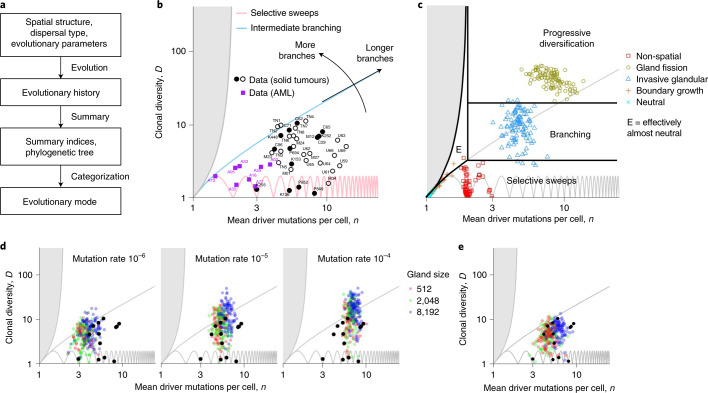
Using summary indices to characterize modes of tumour evolution. **a**, Causal relationships between biological parameters, summary indices and mode of tumour evolution. Tumour architecture, cell dispersal type and other parameters shape the stochastic evolutionary process that gives rise to evolutionary mode. We used evolutionary indices to characterize the modes. **b**, Relationship between clonal diversity *D*, mean driver mutations per cell *n*, and tree topology. Each location within the unshaded region corresponds to a distinct subset of phylogenetic trees. The lower boundary (clonal diversity = 1) corresponds to linear trees in which only one node has size greater than zero (that is, the population comprises only one extant clone). The sequence of pink curves near the lower boundary traces the trajectory of a population that evolves via sequential selective sweeps, so that at any given time, at most two nodes have size greater than zero. The boundary of the shaded region on the left corresponds to star-shaped trees. It is impossible to construct trees for locations within the shaded region. The number of main branches per tree typically increases along anti-clockwise curves between the two boundaries (black arrow). Solid black circles show evolutionary indices derived from multi-region sequencing data for kidney cancers (code suffix K), lung cancers (C) and breast cancers (P). Hollow black circles show evolutionary indices derived from multi-region sequencing data for mesothelioma (M) and single-cell sequencing data for breast cancers (TN) and uveal melanoma (U). Purple squares show evolutionary indices derived from single-cell sequencing data for AML (code suffix A). The pale blue curve corresponds to a particular intermediate degree of branching (Methods and Supplementary information). Patient codes match those in the original publication, except where abbreviated by the following patterns: A02, AML-02-001; C29, CRUK0029; P694, PD9694; M01, MED001; U59, UMM059. **c**, Summary metrics of four example models with different spatial structures and different manners of cell dispersal but identical driver mutation rates and identical driver mutation effects (100 stochastic simulations per model). Neutral counterparts of the four models are represented together as an additional group. Black curves separate four modes of tumour evolution defined in terms of indices *n* and *D* (see also Table 1). Region ‘E’ corresponds to the effectively almost neutral mode. **d**, Evolutionary indices for invasive glandular models with driver fitness effects drawn from an exponential distribution with mean 0.2, and with varied gland size and mutation rate. **e**, Evolutionary indices for an invasive glandular model after adjustment to simulate imperfect sequencing sensitivity (driver mutations with frequency below 5% are removed from the model output). Solid black circles in **d** and **e** are the same as in **b**. Except where specified, parameter values in **c**, **d** and **e** are the same as in Fig. 2.

Together, our models demonstrate that variation in the range of cell–cell interactions and the manner of cell dispersal alone can generate distinct modes of tumour evolution. We next sought to describe these modes more precisely in terms of summary evolutionary indices that can be computed from both our simulations and real cancer genomic data (Fig. 3a). The first index we considered is clonal diversity (denoted *D*), which grows with the number of large nodes in the driver phylogenetic tree (as in the final column of Fig. 2). The second index *n* is the mean number of driver mutations per cell, which represents the average depth of the driver phylogenetic tree. Any pair of values of these two indices corresponds to a distinct set of phylogenetic trees. The nodes of these trees represent clones, and their size is proportional to clone population size. The space of attainable *n* and *D* values (Fig. 3b) is bounded below by the line *D* = 1 and above by the curve *D* = 1/(2−*n*)^2^ (see Methods). Locations close to the upper boundary correspond to more highly branched trees than locations close to the lower boundary, and locations on the left correspond to trees with shorter branches than locations on the right.

To compare our model outcomes to data, we determined the evolutionary indices of phylogenetic trees previously inferred from multi-region sequencing of four solid cancer types: clear cell renal cell carcinoma (ccRCC)^9^, non-small-cell lung cancer (NSCLC)^10^, breast cancer^39^ and mesothelioma^40^. We also calculated indices from single-cell sequencing data for breast cancer^41^ and uveal melanoma^42^. Despite their methodological diversity, these six studies yielded remarkably similar evolutionary indices. The majority of data points (28 of 35) lie above a trajectory corresponding to sequential selective sweeps (pink curve in Fig. 3b) and below a reference curve that represents an intermediate degree of branching (pale blue curve in Fig. 3b and Supplementary information). All the tumours have 1 < *D* < 12 and 3≤*n* < 14. Notwithstanding limitations of sampling, sequencing and phylogenetic inference methods, a useful computational model of invasive tumour evolution should generate summary indices that are consistent with these data points, corresponding to branching evolution with a small number of main branches.

**Extended Data Fig. 5:**
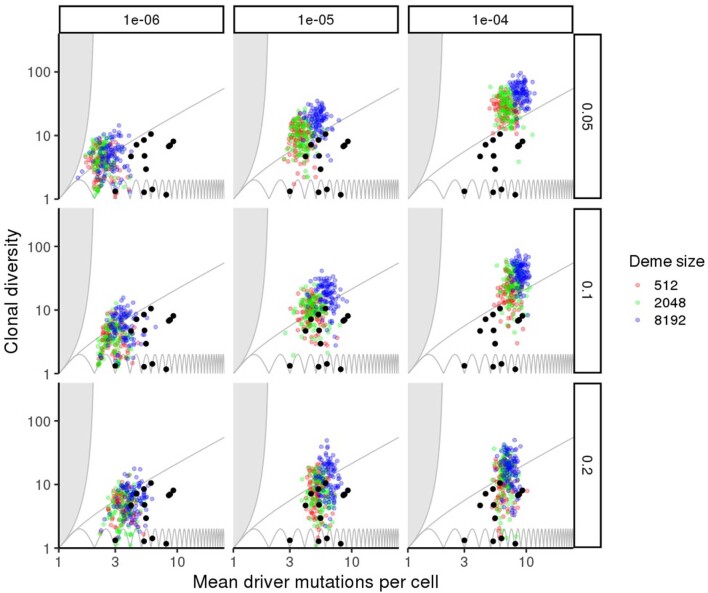
Variation in evolutionary indices *D* and *n* for an invasive glandular model with cell dispersal throughout the tumour and at the tumour boundary. Results are shown for varied gland size (colours), driver mutation rate (columns) and average driver fitness effect (rows), with 100 stochastic simulations per model. Black points show values derived from multi-region sequencing of kidney cancers, lung cancers and breast cancers. Non-varied parameter values are the same as in Fig. 2.

The simulation results of the four models discussed previously form four distinct clusters with respect to the summary indices *n* and *D* (Fig. 3c; mean silhouette width 0.60). Neutral counterparts of these four models—which have the same parameter values, except that the driver fitness effect is reduced to zero—cluster together, near the boundary-growth model. As expected, we find that the evolutionary indices of the solid tumours are consistent with outcomes of our invasive glandular model, and this consistency is robust to varying gland size, driver mutation rate and driver mutation effect within plausible ranges (Extended Data Fig. 5). Particularly close agreement between unadjusted model output and data occurs when the average driver fitness effect is 0.2 (Fig. 3d).

**Extended Data Fig. 6:**
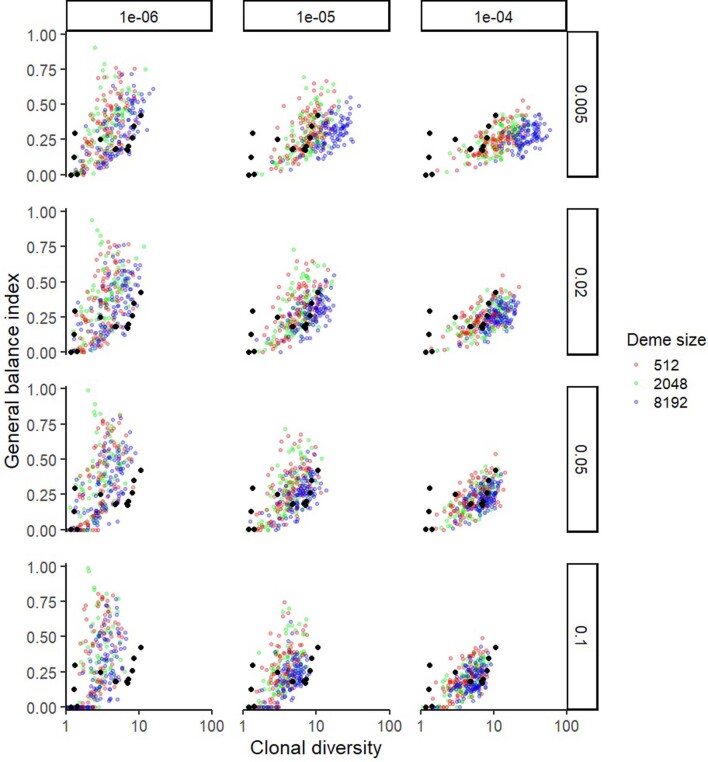
Variation in tree balance index *J*^1^ versus clonal diversity *D* for an invasive glandular model with cell dispersal throughout the tumour and at the tumour boundary. Results are shown for varied gland size (colours), driver mutation rate (columns) and sensitivity threshold (rows), with 100 stochastic simulations per model. Driver mutations with frequency below the sensitivity threshold (0.005, 0.02, 0.05 or 0.1) are removed from the model output before calculating *J*^1^ and *D*. Non-varied parameter values are the same as in Fig. 2. Black points show values derived from multi-region sequencing of kidney cancers, lung cancers and breast cancers.

An important caveat in the above comparison is that the unadjusted model output includes all driver mutations down to a frequency of one in a million, whereas solid tumour sequencing protocols fail to detect most mutations at frequencies below 5%^9^. This difference in sensitivity means that *D* values calculated from data are expected to underestimate true tumour diversity. It follows that a fairer comparison can be made by removing rare mutations from the model output, to simulate imperfect sensitivity. Such adjustment strengthens the agreement between model and data (Fig. 3e and Extended Data Fig. 6).

Since the non-spatial model most plausibly represents liquid tumour evolution, we compared its predictions to additional data for acute myeloid leukaemia^24^. We found robust correspondence between the model and this data set (Fig. 3b,c and Supplementary Fig. 1). Within plausible parameter ranges, 83% of tumours simulated using a non-spatial model have coordinates (*n*, *D*) consistent with the selective-sweeps evolutionary mode.

Alternative models that have different spatial structures are less consistent with data for both solid and liquid tumours. For the gland fission model, 83% of simulated tumours have coordinates above the intermediate-branching curve, corresponding to high values of *D* relative to *n* (Supplementary Fig. 2). For the boundary-growth model, both *n* and *D* are typically close to 1 (Supplementary Fig. 3). These outcomes are summarized in Table 1, which provides quantitative definitions of evolutionary modes in terms of evolutionary indices (see also Fig. 3f and Supplementary Table 1).

**Table 1:** Properties of the four modes of tumour evolution

Evolutionary mode	Role of selection	Definition in terms of summary indices	Tree shape	Associated tumour characteristics	Agreement (%)
Selective sweeps	Strong	*D* < 10/3 and below I-B curve	Approx. linear	Non-spatial (or little spatial structure)	99 (83)
Progressive diversification	Locally strong	*n* > 2; *D* > 20	Highly branched	Gland fission	98 (39)
Branching	Strong but constrained by clonal interference	*n* > 2; 10/3 < *D* < 20	Branched	Invasive glandular (budding; infiltration)	94 (62)
Effectively almost neutral	Weak	*n* < 2 and *D* above I-B curve	Approx. star-shaped	Boundary growth (or very rapid growth)	99 (85)

I-B, intermediate-branching. Ranges of summary indices refer to true values, and it should be noted that values of *D* inferred from multi-region sequencing data will typically underestimate these true values. The ‘Agreement’ column contains the percentage of simulated tumours for which *n* and *D* values conformed to the mode definition (in the third column) when the model possessed the associated tumour characteristics (in the fifth column). For example, in the first row, we give the percentage of tumours simulated using a non-spatial model that conformed to the definition of the selective sweeps mode. The first percentage corresponds to the four non-neutral cohorts of simulations shown in Fig. 3c (one set of parameter values per model). The second percentage (in parentheses) corresponds to the average of multiple cohorts with varied parameter values, as shown in Extended Data Fig. 5 and Supplementary Figs. 1, 2 and 3. Additional results are given in Supplementary Table 1.

Results for a variant of the invasive glandular model, in which normal cells are absent and the tumour grows into empty space, are also less consistent with data (Supplementary Fig. 4). In this empty-space model, the speed at which the tumour expands (via cell dispersal into empty space) typically exceeds the speed at which clones spread within the tumour (via cell dispersal into fully occupied glands), which leads to a more star-shaped or highly branched phylogeny (high *D* relative to *n*). Conversely, when tumour cells must compete with normal cells at the tumour boundary (as in the third row of Fig. 2), the speed at which driver mutations spread within the tumour is similar to the speed of tumour growth, which enables some driver mutations to reach high frequency and results in sparser branching (Extended Data Fig. 5). Yet another alternative model, which includes normal cells but confines cell dispersal to the tumour boundary, thwarts the spread of driver mutations and generates similar *D* but smaller *n* values (Supplementary Fig. 5).

### Further analysis of tumour evolutionary modes

A complementary way to describe modes of tumour evolution is in terms of phylogenetic tree shape or balance. Because tree balance indices developed for characterizing organismal evolution are poorly suited to tumour data, we developed an index^43^ that is robust to variation in sampling and sequencing protocols (Methods). This index *J*^1^ takes a high value for trees in which branching events tend to split the tree into subtrees of similar size. Low values are assigned to trees that are approximately linear or are dominated by a single node.

**Fig. 4:**
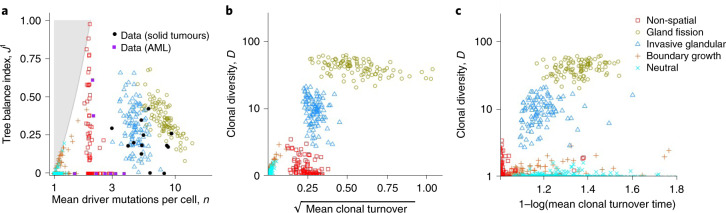
Alternative summary indices for characterizing modes of tumour evolution. Index values are shown for four models representative of their evolutionary modes with different spatial structures and different manners of cell dispersal but identical driver mutation rates and identical driver mutation effects (100 stochastic simulations per model). Neutral counterparts of the four models are represented together as an additional group. **a**, Tree balance *J*^1^ versus mean number of driver mutations per cell *n*. Solid black circles show evolutionary indices derived from multi-region sequencing data. Solid purple squares show values derived from single-cell sequencing data for acute myeloid leukaemia. Hollow coloured circles are the predictions of the four models and their neutral counterparts, excluding mutations with frequency below 1%. It is impossible to construct trees for locations within the shaded region. **b**, Clonal diversity *D* versus mean clonal turnover $\overline{{{\Theta }}}$. **c**, Clonal diversity *D* versus mean clonal turnover time ${\overline{T}}_{{{\Theta }}}$. Both the mean clonal turnover index and the mean clonal turnover time have been transformed to equalize cluster widths and facilitate comparison between plots. In **c**, low *x*-axis values indicate that most clonal turnover occurred late in tumour growth.

**Extended Data Fig. 7:**
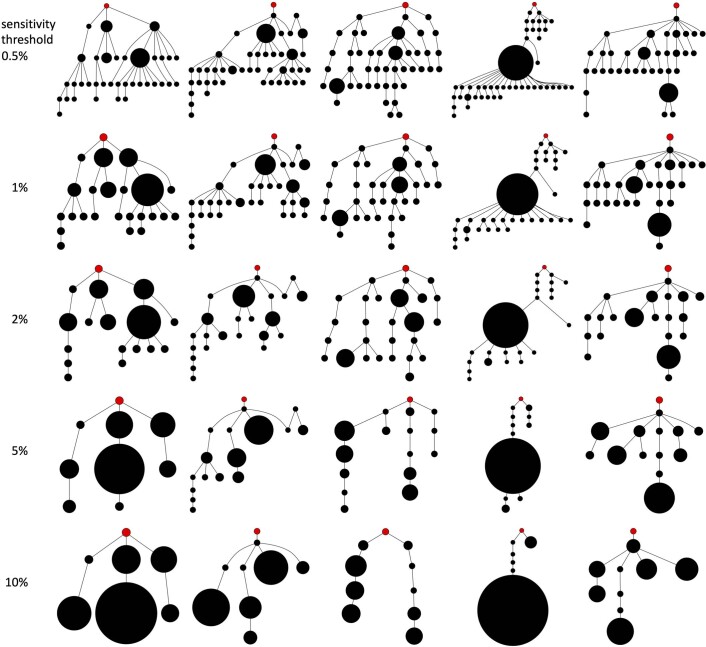
Driver phylogenetic trees resulting from an evolutionary model with cell dispersal throughout the tumour and at the tumour boundary (512 cells per gland). The rows show final outcomes of the same five simulations (in the same order) after adjustment to simulate different sensitivities in detecting rare mutations. Driver mutations with frequency below the sensitivity threshold are removed from the model output. This means that if the combined frequency of a clone and its descendants is below the sensitivity threshold then the clone is merged with its parent clone. The sensitivity threshold is varied from 0.5% (top row) to 10% (bottom row). Node size corresponds to clone population size and the founding clone is coloured red.

**Extended Data Fig. 8:**
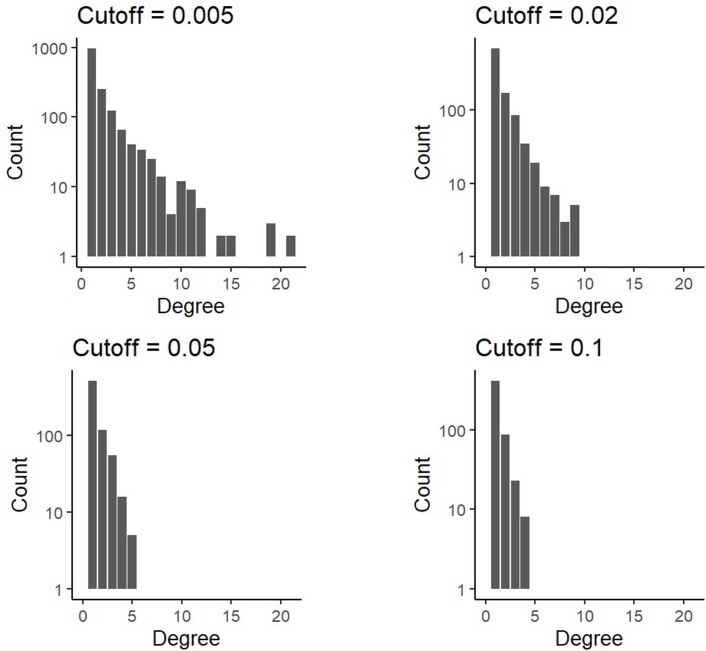
Combined degree distribution for 100 driver phylogenetic trees resulting from an evolutionary model with cell dispersal throughout the tumour and at the tumour boundary (512 cells per gland). The four panels correspond to the same set of simulations after adjustment to simulate different sensitivities in detecting rare mutations. Driver mutations with frequency below the sensitivity threshold are removed from the model output. This means that if the combined frequency of a clone and its descendants is below the sensitivity threshold then the clone is merged with its parent clone. The sensitivity threshold is varied from 0.5% to 10%.

Just as for indices *n* and *D*, the tree balance values predicted by our invasive glandular tumour model are consistent with the values obtained from sequencing data (Fig. 4a). Typical *J*^1^ values for both this model and the data are between 0 and 0.5—substantially below the maximum value of 1 corresponding to perfectly balanced trees. The consistency remains when we adjust the model output by removing rare driver mutations (Extended Data Fig. 6, which constitutes a fairer comparison), even though the associated trees appear very different (Extended Data Fig. 7) and have dissimilar degree distributions (Extended Data Fig. 8). Agreement between model and data is also observed for alternative balance indices after removing rare mutations (Supplementary Figs. 6, 7 and 8). Conversely, neutral models and models that do not account for glandular structure predict smaller or more variable tree balance values than the data for solid tumours (Fig. 4a). Tree balance values for the non-spatial model are consistent with data for acute myeloid leukaemia (Fig. 4a).

**Extended Data Fig. 9:**
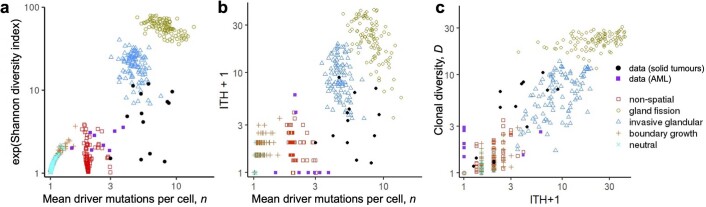
Alternative diversity indices. a, Variation in the exponential of the Shannon diversity index versus mean number of driver mutations per cell (*n*). b, Variation in the ITH index versus mean number of driver mutations per cell (*n*). c, Correlation between the inverse Simpson index (*D*) and the ITH index. Coloured points correspond to four example models with different spatial structures and different manners of cell dispersal but identical driver mutation rates and identical driver mutation effects (100 stochastic simulations per model). Neutral counterparts of the four models are represented together as an additional group. Mutations with frequency less than 1% are removed from model outcomes before calculating ITH and *D*. Black circular points show values derived from multi-region sequencing of kidney cancers, lung cancers and breast cancers. Purple squares show values derived from single-cell sequencing data for acute myeloid leukaemia. Estimates of the Shannon index and ITH index based on multi-region sequencing data are expected to be lower than true values because these indices are sensitive to the removal of rare types, many of which are likely to be missing from the data due to sampling error.

Whereas *n*, *D* and *J*^1^ are determined only by the final tumour state, other indices can be based on time series data. For example, the mean clonal turnover magnitude $\overline{{{\Theta }}}$ provides an alternative to *n* for measuring the extent of evolutionary change, and the mean clonal turnover time ${\overline{T}}_{{{\Theta }}}$ indicates whether evolutionary change occurs mostly early or late during tumour growth (Methods). As expected, after an appropriate axis transformation, the pattern of clusters of *D* versus $\overline{{{\Theta }}}$ (Fig. 4b) resembles the pattern of clusters of *D* versus *n* (Fig. 3c). Plotting *D* versus a transformed ${\overline{T}}_{{{\Theta }}}$ reveals a somewhat similar pattern, except that models with low $\overline{{{\Theta }}}$ exhibit high stochastic variation in ${\overline{T}}_{{{\Theta }}}$ (Fig. 4c). Clonal turnover occurs relatively late in the non-spatial model but throughout tumour growth in the gland fission model. Given sufficient data, evolutionary modes can thus be described and classified in terms of various summary indices capturing distinct aspects of tumour evolution (for alternative diversity indices, see Extended Data Fig. 9).

### Influence of tumour architecture on mutation frequency distributions

**Fig. 5:**
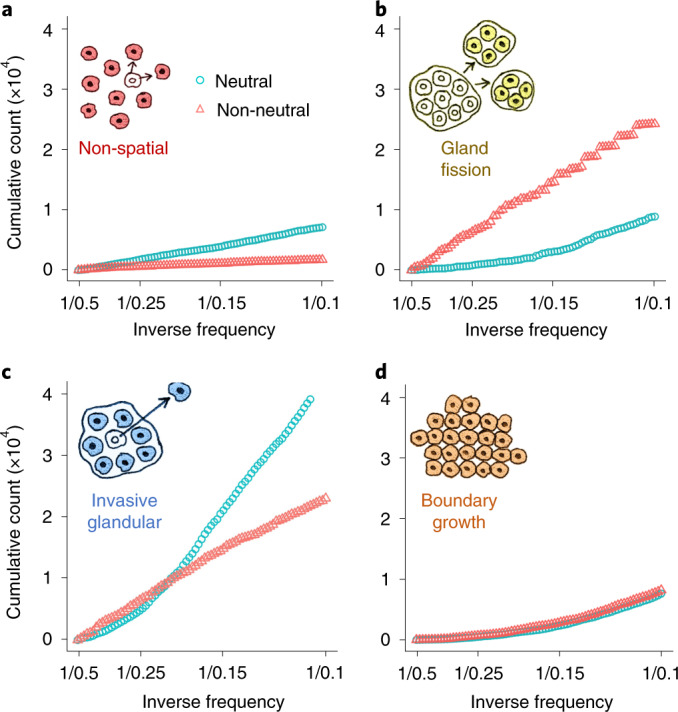
Mutation frequency distributions predicted by our model. **a**–**d**, Mutation frequency distributions for simulations with only neutral mutations (blue circles) or both neutral and driver mutations (red triangles). Cumulative mutation count is plotted against inverse mutation frequency (1/*f*), restricted to mutations with frequencies between 0.1 and 0.5. Each distribution represents combined data from 100 simulations.

As researchers and clinicians seldom have access to multi-regional sequencing data, or the longitudinal data needed to track how tumour clone sizes change over time, tumour phylogenies and evolutionary parameters are commonly inferred from mutation frequencies measured from a single biopsy sampled at a single time point. Moreover, current cancer sequencing technologies are neither sensitive enough to detect the majority of low-frequency mutations, nor precise enough to distinguish between high-frequency and clonal (100% frequency) mutations. Accordingly, the most relevant part of the mutation frequency distribution for practical purposes is in the intermediate frequency range. One way to examine differences between distributions within this intermediate range is to plot the cumulative mutation count (the number of mutations present at or above frequency *f*) versus the inverse mutation frequency (1/*f*). In a neutral non-spatial model, this graph is a straight line (Fig. 5a, blue points). Because the transformed mutation frequency distributions of many human cancers are also approximately linear, it has been proposed that neutral tumour evolution is widespread^12^. Deviations from this theoretical straight line have been taken as evidence of selection^27,44^.

**Extended Data Fig. 10:**
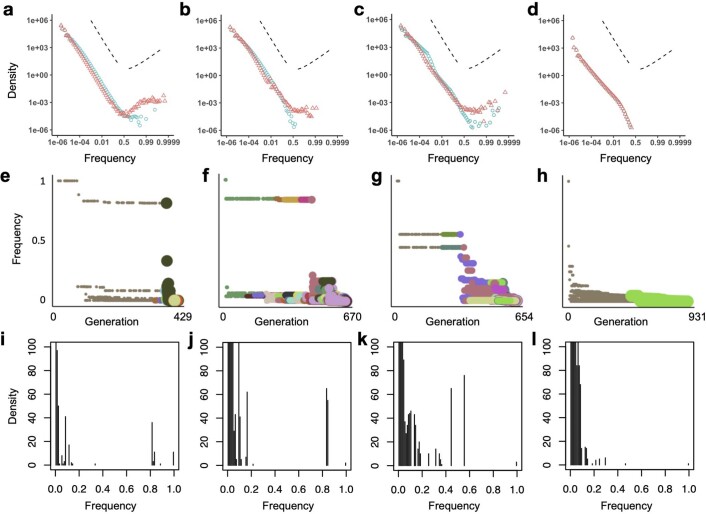
Mutation frequency distributions for simulated tumours. a-d, Complete mutation frequency distributions for models with only neutral mutations (blue points) or both neutral and driver mutations (red points). Each distribution represents combined data from 100 simulations of each of the four model types of Figs. 2 and 3. To clarify the shape of the distributions, especially at high frequencies, the x-axes are transformed as logit(*x*) = log(*x*/(1 − *x*)), which is approximately equal to log *x* when *x* is much less than 1. Dashed lines indicate the slope predicted for an exponentially-growing population acquiring only neutral mutations (negative slope) and a prediction of the Bolthausen-Sznitman coalescent (positive slope), which has been shown to describe genealogies when a constant-size population expands into uninhabited territory^92^ or when a constant-size population acquires both neutral and highly beneficial mutations^93^. e-h, Mutation frequency versus timing of mutation for the specific model instances of Fig. 2. Point colour corresponds to clone (as in Fig. 2), and size corresponds to the division rate of cells within the clone. Driver mutations are typically preceded by a string of hitchhiking passenger mutations with similar frequencies. This figure format is inspired by Figure 2 of ref. ^4^. i-l, Mutation frequency distributions for the specific model instances of Fig. 2, with linear axes. Results are shown for a non-spatial branching process (a, e, i); tumour growth via gland fission (b, f, j); cell dispersal throughout the tumour and at the tumour boundary (c, g, k); and a boundary-growth model (d, h, l). Parameter values are the same as in Figs. 2 and 3.

Our population genetics modelling illustrates how not only selection but also tumour architecture has important effects on tumour mutation frequency distributions (Fig. 5 and Extended Data Fig. 10). In particular, when the cumulative mutation count is plotted against the inverse mutation frequency, the curve for the neutral model is no longer linear. Instead, for spatial models, the average non-neutral curve can be closer to a straight line than the average neutral model curve. These results confirm and extend previous findings^27,35^ indicating that methods using mutation frequencies to infer selection in solid tumours can yield incorrect conclusions if they fail to account for effects of population structure. Inappropriate choice of null model can therefore explain otherwise contradictory findings regarding the prevalence of neutral evolution in human cancers^13,45^.

## Discussion

In summary, we have found that differences in the range of cell–cell interactions and the manner of cell dispersal are sufficient to generate a spectrum of tumour evolutionary modes. This finding has important implications both for understanding tumour genomic data and for interpreting the results of previous computational models. Whereas mathematical oncologists have focused on mutation fitness effects^5,12,27,44,46,47^ or microenvironmental heterogeneity^15^, our perspective instead emphasizes the importance of population structure and gene flow in tumour evolution.

Prominent studies have variously used non-spatial models^12,21,44,46^, gland fission models^5,27^ or variants of the Eden growth model (in which cells compete with their nearest neighbours)^32,47^ to investigate aspects of tumour evolution (see Methods for further discussion of previous work). Our results imply that, at best, each of these model types is appropriate only in special cases. Accurate models of solid tumour evolution must faithfully recapitulate interactions within localized patches of cancer cells—the so-called tumour communities^29^—and between cancer cells and normal cells.

Consistent with previous work^48^, our models predict substantial variability in tumour evolutionary modes due to stochasticity in the timing, location and fitness effects of driver mutations. Our finding that this random variation approaches the variability observed within and between solid tumour types (Fig. 3b,c) suggests that it will be challenging to infer precise information about tumour structure and growth patterns from phylogenetic data, even given previous knowledge of mutation rates and fitness effects. Nevertheless, of the model types we have examined, we have shown that the most plausible for simulating evolution in the majority of malignant solid tumours, which exhibit branching evolution^11^, is the invasive glandular model introduced herein. A key feature of this model is that the speed at which a fitter clone spreads within its immediate ancestor is similar to the ancestor’s own expansion speed.

It follows from our findings that tumour architecture determines how well biopsy samples reflect intratumour heterogeneity. Oncologists typically base treatment decisions on the presence or absence of particular mutations in cells taken from only a small region of a solid tumour. Tumour types with structures that promote diversification are predicted to be the least responsive to targeted therapies, unless truncal mutations can be reliably identified and targeted.

Our framework also implies that a change in tumour architecture during cancer progression can lead to a change in the mode of tumour evolution. For example, the ‘big bang’ model of colorectal cancer^4,5^ posits that early selective sweeps are followed by effectively neutral evolution, such that mutation frequency is determined by the time of mutation occurrence. This idea was previously examined using a computational model of tumour growth via gland fission, with a maximum of one driver mutation per cell^5^. Based on more sophisticated population genetics modelling, we find reason to expect ongoing selection throughout the very early stages of colorectal tumour progression (when growth is driven by gland fission), enabling multiple driver mutations to reach high frequencies. In later stages, after cells from the adenoma invade neighbouring tissue and give rise to an adenocarcinoma, we predict a transition to either branching evolution or – because the invasion begins with numerous and/or highly transformed, rapidly expanding subclones^49^ – effectively neutral evolution. Punctuated evolution in colorectal tumours can thus be explained by the transition from gland fission to invasive growth. This explanation is broadly consistent with the big bang model and more recent multi-region sequencing studies^6,49,50^, while also agreeing with results of comparative genomic analysis, which indicate that colorectal cancers evolve subject to strong positive selection and have more driver mutations per cell than most other cancer types^13,51^. Transitions between evolutionary modes were recently investigated in a mathematical modelling study of ductal carcinoma,^52^ which complements the current work by likewise highlighting the importance of spatial competition.

In clear cell renal cell carcinoma, separate studies have found that tumour architecture^53,54^ and evolutionary trajectory^9^ are predictors of cancer progression and survival. Evolutionary mode correlates with both tumour architecture and clinical outcome in childhood cancers^8,55^. The manner of cell dispersal has prognostic value in colorectal and other solid cancers^28^. By mechanistically connecting tumour architecture to the mode of tumour evolution, our work provides a blueprint for a new generation of patient-specific models for forecasting tumour progression^16,48^ and for optimizing evolutionarily-informed treatment regimens^56–58^. This drive towards personalized models motivates further efforts to characterize how spatial structure interacts with other biological factors, such as spatially-varying carrying capacity^29^, alternative manners of cell dispersal^19^, immune interactions^59^, cancer stem cell hierarchies^60^, and frequency- or density-dependent cell fitness.^17^

## Methods

### Previous mathematical models of tumour population genetics

Many previous studies of tumour population genetics have used non-spatial branching processes^21^, in which cancer clones grow exponentially without interacting. Unless driver mutations increase cell fitness by less than 1%, these models predict lower clonal diversity and lower numbers of driver mutations than typically observed in solid tumours^46^. Among spatial models, a popular option is the Eden growth model (or boundary-growth model), in which cells are located on a regular grid with a maximum of one cell per site, and a cell can divide only if an unoccupied neighbouring site is available to receive the new daughter cell^32,47,61^. Other methods with one cell per site include the voter model^32,62,63^ (in which cells can invade neighbouring occupied sites) and the spatial branching process^47^ (in which cells budge each other to make space to divide). Further mathematical models have been designed to recapitulate glandular tumour structure by allowing each grid site or ‘deme’ to contain multiple cells and by simulating tumour growth via deme fission throughout the tumour^5,26^ or only at the tumour boundary^27^. A class of models in which cancer cells are organized into demes and disperse into empty space has also been proposed^36,52,64^. Supplementary Table 2 summarizes selected studies representing the state of the art of stochastic modelling of tumour population genetics.

Our main methodological innovations are to implement all these distinct model structures, and additional models of invasive tumours, within a common framework, and to combine them with methods for tracking driver and passenger mutations at single-cell resolution. The result is a highly flexible framework for modelling tumour population genetics that can be used to examine consequences of variation not only in mutation rates and selection coefficients, but also in spatial structure and manner of cell dispersal^65^.

### Computational model structure

Simulated tumours in our models are made up of patches of interacting cells located on a regular grid of sites. In keeping with the population genetics literature, we refer to these patches as demes. All demes within a model have the same carrying capacity, which can be set to any positive integer. Each cell belongs to both a deme and a genotype. If two cells belong to the same deme and the same genotype then they are identical in every respect, and hence the model state is recorded in terms of such subpopulations rather than in terms of individual cells. For the sake of simplicity, computational efficiency and mathematical tractability, we assume that cells within a deme form a well-mixed population. The well-mixed assumption is consistent with previous mathematical models of tumour evolution^5,26,27,36,64^ and with experimental evidence in the case of stem cells within colonic crypts^66^.

### Initial conditions

A simulation begins with a single tumour cell located in a deme at the centre of the grid. If the model is parameterized to include normal cells, then these are initially distributed throughout the grid such that each deme’s population size is equal to its carrying capacity. Otherwise, if normal cells are absent, then the demes surrounding the tumour are initially unoccupied.

### Stopping condition

The simulation stops when the number of tumour cells reaches a threshold value. Because we are interested only in tumours that reach a large size, if the tumour cell population succumbs to stochastic extinction, then results are discarded and the simulation is restarted (with a different seed for the pseudo-random number generator).

### Within-deme dynamics

Tumour cells undergo stochastic division, death, dispersal and mutation events, whereas normal cells undergo only division and death. The within-deme death rate is density-dependent. When the deme population size is less than or equal to the carrying capacity, the death rate takes a fixed value *d*_0_ that is less than the initial division rate. When the deme population size exceeds carrying capacity, the death rate takes a different fixed value *d*_1_ that is much greater than the largest attainable division rate. Hence, all genotypes grow approximately exponentially until the carrying capacity is attained, after which point the within-deme dynamics resemble a birth–death Moran process—a standard, well characterized model of population genetics.

In all spatially structured simulations, we set *d*_0_ = 0 to prevent demes from becoming empty. For the non-spatial (branching process) model, we set *d*_0_ > 0 and dispersal rate equal to zero, so that all cells always belong to a single deme (with carrying capacity greater than the maximum tumour population size).

### Mutation

When a cell divides, each daughter cell inherits its parent’s genotype plus a number of additional mutations drawn from a Poisson distribution. Each mutation is unique, consistent with the infinite-sites assumption of canonical population genetics models. Whereas some previous studies have examined the effects of only a single driver mutation (Supplementary Table 2), in our model there is no limit on the number of mutations a cell can acquire. Most mutations are passenger mutations with no phenotypic effect. The remainder are drivers, each of which increases the cell division or dispersal rate.

The programme records the immediate ancestor of each clone (defined in terms of driver mutations) and the matrix of Hamming distances between clones (that is, for each pair of clones, how many driver mutations are found in only one clone), which together allow us to reconstruct driver phylogenetic trees. To improve efficiency, the distance matrix excludes clones that failed to grow to more than ten cells and failed to produce any other clone before becoming extinct.

### Driver mutation effects

Whereas previous models have typically assumed that the effects of driver mutations combine multiplicatively, this can potentially result in implausible trait values (especially in the case of division rate if the rate of acquiring drivers scales with the division rate). To remain biologically realistic, our model invokes diminishing returns epistasis, such that the average effect of driver mutations on a trait value *r* decreases as *r* increases. Specifically, the effect of a driver is to multiply the trait value *r* by a factor of 1 + *s*(1 − *r*/*m*), where *s* > 0 is the mutation effect and *m* is an upper bound. Nevertheless, because we set *m* to be much larger than the initial value of *r*, the combined effect of drivers in all models in the current study is approximately multiplicative. For each mutation, the value of the selection coefficient *s* is drawn from an exponential distribution.

### Dispersal

Depending on model parameterization, dispersal occurs via either invasion or deme fission (Supplementary Table 3). In the case of invasion, the dispersal rate corresponds to the probability that a cell newly created by a division event will immediately attempt to invade a neighbouring deme. This particular formulation ensures consistency with a standard population genetics model known as the spatial Moran process. The destination deme is chosen uniformly at random from the four nearest neighbours (von Neumann neighbourhood). Invasion can be restricted to the tumour boundary, in which case the probability that a deme can be invaded is 1 − *N*/*K* if *N*≤*K* and 0 otherwise, where *N* is the number of tumour cells in the deme and *K* is the carrying capacity. If a cell fails in an invasion attempt, then it remains in its original deme. If invasion is not restricted to the tumour boundary, then invasion attempts are always successful.

In fission models, a deme can undergo fission only if its population size is greater than or equal to carrying capacity. As with invasion, deme fission immediately follows cell division (so that results for the different dispersal types are readily comparable). The probability that a deme will attempt fission is equal to the sum of the dispersal rates of its constituent cells (up to a maximum of 1). Deme fission involves moving half of the cells from the original deme into a new deme, which is placed beside the original deme. If the dividing deme contains an odd number of cells, then the split is necessarily unequal, in which case each deme has a 50% chance of receiving the larger share. Genotypes are redistributed between the two demes without bias according to a multinomial distribution. Cell division rate has only a minor effect on deme fission rate because a deme created by fission takes only a single cell generation to attain carrying capacity.

If fission is restricted to the tumour boundary, then the new deme’s assigned location is chosen uniformly at random from the four nearest neighbours, and if the assigned location already contains tumour cells, then the fission attempt fails. If fission is allowed throughout the tumour, then an angle is chosen uniformly at random, and demes are budged along a straight line at that angle to make space for the new deme beside the original deme.

Our particular method of cell dispersal was chosen to enable comparison between our results and those of previous studies and to facilitate mathematical analysis. In particular, when the deme carrying capacity is set to 1, our model approximates an Eden growth model (if fission is restricted to the tumour boundary, or if dispersal is restricted to the tumour boundary and normal cells are absent), a voter model (if invasion is allowed throughout the tumour) or a spatial branching process (if fission is allowed throughout).

To fairly compare different spatial structures and manners of cell dispersal, we set dispersal rates in each case such that the time taken for a tumour to grow from one cell to one million cells is approximately the same as in the neutral Eden growth model with maximal dispersal rate. This means that, across models, the cell dispersal rate decreases with increasing deme size. Given that tumour cell cycle times are on the order of a few days, the timespans of several hundred cell generations in our models realistically correspond to several years of tumour growth. More specifically, if we assume tumours take between 5 and 50 years to grow and the cell cycle time is between 1 and 10 days (both uniform priors), then the number of cell generations is between 400 and 8,000 in 95% of plausible cases. This order of magnitude is consistent with tumour ages inferred from molecular data^67^.

We note that, in addition to gland fission, gland fusion has been reported in normal human intestine^68^, which raises the possibility that gland fusion could occur during colorectal tumour development. However, the rate of crypt fission in tumours is much elevated relative to the rate in healthy tissue, and must exceed the rate of crypt fusion (or else the tumour would not grow). Therefore, even if crypt fusion occurs in human tumours, we do not expect it to have a substantial influence on evolutionary mode. This view is supported by previous computational modelling^69^.

### Two versus three dimensions

We chose to conduct our study in two dimensions for two main reasons. First, the effects of deme carrying capacity on evolutionary dynamics are qualitatively similar in two and three dimensions, yet a two-dimensional model is simpler, easier to analyse, and easier to visualize. Second, we aimed to create a method that is readily reproducible using modest computational resources and yet can represent the long-term evolution of a reasonably large tumour at single-cell resolution.

One million cells in two dimensions corresponds to a cross-section of a three-dimensional tumour with many more than one million cells. Therefore, compared to a three-dimensional model, a two-dimensional model can provide richer insight into how evolutionary dynamics change over a large number of cell generations. Developing an approximate, coarse-grained analogue of our model that can efficiently simulate the population dynamics of very large tumours with different spatial structures in three dimensions is an important direction for future research.

### Implementation

The programme implemented Gillespie’s exact stochastic simulation algorithm^70^ for statistically correct simulation of cell events. The order of event selection is (1) deme, (2) cell type (normal or tumour), (3) genotype, and (4) event type. At each stage, the probability of selecting an item (deme, cell type, genotype or event type) is proportional to the sum of event rates for that item, within the previous item. We measured elapsed time in terms of cell generations, where a generation is equal to the expected cell cycle time of the initial tumour cell.

### Sequencing data

We surveyed the multi-region and single-cell tumour sequencing literature to identify data sets suitable for comparison with our model results. Studies published before 2015 (for example, refs. ^71–74^) were excluded as they were found to have insufficient sequencing depth for our purposes. We also excluded studies that reconstructed phylogenies using samples from metastases or from multifocal tumours (for example, refs. ^75–80^) because our model is not designed to correspond to such scenarios. The seven studies we chose to include in our comparison are characterized by either high-coverage multi-region sequencing or large-sample single-cell sequencing of several tumours.

The ccRCC investigation^81^ we selected involved multi-region deep sequencing, targeting a panel of more than 100 putative driver genes. Three studies of NSCLC^10^, mesothelioma^40^ and breast cancer^39^ conducted multi-region whole-exome sequencing (first two studies) or whole-genome sequencing (latter study), and reported putative driver mutations. We also used data from single-cell RNA sequencing studies of uveal melanoma^42^ and breast cancer^41^, in which chromosome copy number variations were used to infer clonal structure, and a study of acute myeloid leukaemia (AML) that used single-cell DNA sequencing^24^. All seven studies constructed phylogenetic trees, which are readily comparable to the trees predicted by our modelling. The methodological diversity of these studies contributes to demonstrating the robustness of the patterns we seek to explain.

From each of the seven cohorts, we obtained data for between three and eight tumours. In the ccRCC data set, we focused on the five tumours for which driver frequencies were reported in the original publication. For NSCLC, we used data for the five tumours for which at least six multi-region samples were sequenced. In mesothelioma, we selected the six tumours that had at least five samples taken. From the breast cancer multi-region study, we used data for the three untreated tumours that were subjected to multi-region sequencing. From the single-cell sequencing studies of uveal melanoma and breast cancer, we used all the published data (eight tumours in each case), and from the AML study, we selected a random sample of eight tumours.

In multi-region sequencing data sets, it is uncertain whether all putative driver mutations were true drivers of tumour progression. One way to interpret the data (interpretation I1) is to assume that all putative driver mutations were true drivers that occurred independently. Alternatively, the more conservative interpretation I2 assumes that each mutational cluster (a distinct peak in the variant allele frequency distribution) corresponds to exactly one driver mutation, while all other mutations are hitchhikers. Thus, I1 permits linear chains of nodes that in I2 are combined into single nodes (compare Supplementary Figs. 9 and 10), and I1 leads to a higher estimate of the mean number of driver mutations per cell (our summary index *n*). If both the fraction of putative driver mutations that are not true drivers (false positives) and the fraction of true driver mutations that are not counted as such (false negatives) are low, or if these fractions approximately cancel out, then interpretation I1 will give a good approximation of *n* whereas I2 will give a lower bound. For the ccRCC, NSCLC and breast cancer cases in our data set, I1 generates values of *n* in the range 3–10 (mean 6.1), consistent with estimates based on other methodologies^13,51^, whereas for I2 the range is only 1–4 (mean 2.5). Accordingly, we used interpretation I1.

### Clonal diversity index

To measure clonal diversity, we used the inverse Simpson index defined as $D=1/{\sum }_{i}{p}_{i}^{2}$, where *p*_*i*_ is the frequency of the *i*th combination of driver mutations. For example, if the population comprises *k* clones of equal size, then *p*_*i*_ = 1/*k* for every value of *i*, and so *D* = 1/(*k* × 1/*k*^2^) = *k*. Clonal diversity has a lower bound *D* = 1. The inverse Simpson index is relatively robust to adding or removing rare types, which makes it appropriate for comparing data sets with differing sensitivity thresholds. Further examples are illustrated in Supplementary Fig. 11.

*D* is constrained by an upper bound for trees with *n* < 2, where *n* is the mean number of driver mutations per cell. Indeed, *n* = ∑_*i*_*ip*_*i*_ ≥ *p*_1_ + 2(1 − *p*_1_) = 2 − *p*_1_, so *p*_1_ ≥ 2 − *n* > 0, since *n* < 2. Therefore,

$$D=\frac{1}{{\sum }_{i}{p}_{i}^{2}}\le \frac{1}{{p}_{1}^{2}}\le \frac{1}{{(2-n)}^{2}}.$$

To see that this bound is tight, assume 1 ≤ *n* < 2 and consider a star-shaped tree with *N* nodes such that *p*_1_ = 2 − *n* and other nodes have equal weights *p*_*i*_ = (1 − *p*_1_)/(*N* − 1) = (*n* − 1)/(*N* − 1) for *i* ≥ 2. The mean number of driver mutations per cell is *p*_1_ + 2(1 − *p*_1_) = 2 − *p*_1_ = *n*, and the inverse Simpson index is

$$\begin{array}{l}D=\frac{1}{\mathop{\sum }\nolimits_{i = 1}^{N}{p}_{i}^{2}}=\frac{1}{{p}_{1}^{2}+\mathop{\sum }\nolimits_{i = 2}^{N}{p}_{i}^{2}}\\=\frac{1}{{(2-n)}^{2}+(N-1){((n-1)/(N-1))}^{2}}=\frac{1}{{(2-n)}^{2}+{(n-1)}^{2}/(N-1)}.\end{array}$$

This quantity goes to 1/(2 − *n*)^2^ as the number of nodes *N* goes to infinity, so the bound 1/(2 − *n*)^2^ may be approached arbitrarily closely.

It is informative to derive the relationship between *D* and *n* for a population that evolves via a sequence of clonal sweeps, such that each new sweep begins only after the previous sweep is complete. For a given value of *n*, our simulations rarely produce trees with *D* values below the curves of this trajectory. Suppose that a population comprises a parent type and a daughter type, with frequencies *p* and 1 − *p*, respectively. If the daughter has *m* driver mutations, then the parent must have *m* − 1 driver mutations and *n* must satisfy *m* − 1 ≤ *n* ≤ *m*. More specifically,

$$n=(m-1)p+m(1-p)=m-p\ \Rightarrow \ p=m-n=1-\{n\},$$

where {*n*} denotes the fractional part of *n* (or 1 if *n* = *m*). The trajectory is therefore described by

$$D=\frac{1}{{p}^{2}+{(1-p)}^{2}}=\frac{1}{{(1-\{n\})}^{2}+{\{n\}}^{2}}.$$

We additionally calculated a curve representing the maximum possible diversity of linear trees. In the main text and below, we refer to this curve as corresponding to trees with an intermediate degree of branching. Specifically, this intermediate-branching curve is defined such that for every point below the curve (and with *D* > 1), there exist both linear trees and branching trees that have the corresponding values of *n* and *D*, whereas for every point above the curve there exist only branching trees. Derivation of the curve’s equation is provided in Supplementary Information. A first-order approximation (accurate within 1% for *n* ≥ 2.2) is *D* ≈ 9(2*n* − 1)/8.

To assess the extent to which clusters of points (*n*, *D*) are well separated, we calculated silhouette widths using the cluster R package^82^. A positive mean silhouette width indicates that clusters are distinct.

### Other diversity indices

Our diversity index fulfills the same purpose as the intratumour heterogeneity (ITH) index used in the TRACERx Renal study^9^, defined as the ratio of the number of subclonal driver mutations to the number of clonal driver mutations. However, compared to ITH, our index has the advantages of being a continuous variable and being robust to methodological differences that affect ability to detect low-frequency mutations. In calculating ITH from sequencing data, we included all putative driver mutations, whereas ref. ^9^ used only a subset of mutations. For model output, we classified mutations with frequency above 99% as clonal and we excluded mutations with frequency less than 1%. ITH and the inverse Simpson index are strongly correlated across our models (Spearman’s *ρ* = 0.98, or *ρ* = 0.81 for cases with *D* > 2; Extended Data Fig. 9c).

The Shannon index, defined as ${\sum }_{i}{p}_{i}{{\mathrm{log}}}\,{p}_{i}$, is another alternative to the Simpson index. The exponential of this index has the same units as the inverse Simpson index (equivalent number of types). Compared to the Simpson index, the Shannon index gives more weight to rare types, which makes it somewhat less suitable for comparing data sets with differing sensitivity thresholds.

### Defining evolutionary modes in terms of indices *D* and *n*

In defining regions in terms of indices *D* and *n* (Table 1 and Fig. 3c), we first noted that if a population undergoes a succession of non-overlapping clonal sweeps, then at most two clones coexist at any time, and hence *D* ≤ 2. Allowing for some overlap between sweeps, we defined the ‘selective sweeps’ region as having *D* < 10/3 and *D* below the intermediate-branching curve. We put the upper boundary at *D* = 10/3 because this intersects with the intermediate-branching curve at *n* = 2.

We used *D* = 20 to define the boundary between the ‘branching’ and ‘progressive diversification’ regions. The TRACERx Renal study^9^ instead categorized trees containing more than 10 clones as highly branched, as opposed to branched. It is appropriate for us to use a higher threshold because our regions are based on true tumour diversity values, rather than the typically lower values inferred from multi-region sequencing data. Finally, we defined an ‘effectively almost neutral’ region containing star-shaped trees with *n* < 2 and *D* above the intermediate-branching curve.

It is possible to construct trees that do not fit the labels we have assigned to regions. For example (as shown in Supplementary Information), there exist linear trees within the branching and progressive diversification regions. Such exceptions are an unavoidable consequence of representing high-dimensional objects, such as phylogenetic trees, in terms of a small number of summary indices. Our labels are appropriate for the subset of trees that we have shown to arise from tumour evolution.

### Previously defined tree balance indices

Conventionally, the balance of a tree is the degree to which branching events split the tree into subtrees with the same number of leaves, or terminal nodes. A balanced tree thus indicates more equal extinction and speciation rates than an unbalanced tree^83^. Tree balance indices are commonly used to assert the correctness of tree reconstruction methods and to classify trees. We considered three previously defined indices, all of which are imbalance indices, which means that more balanced trees are assigned smaller values. We subtracted each of these indices from 1 to obtain measurements of tree balance.

Let *T* = (*V*, *E*) be a tree with a set of nodes *V* and edges *E*. Let ∣*V*∣ = *N*, and hence ∣*E*∣ = *N* − 1 (since each node has exactly one parent, except the root). We defined *l* as the number of leaves of the tree. The root is labelled 1 and the leaves are numbered from *N* − *l* + 1 to *N*. There is only one cladogram with two leaves, which is maximally balanced according to all the previously defined indices discussed below. We also considered the single-node tree to be maximally balanced with respect to these previously defined indices. The following definitions then apply when *l* ≥ 3.

For each leaf *j*, we defined *ν*_*j*_ as the number of interior nodes between *j* and the root, which is included in the count. Then a normalized version of Sackin’s index, originally introduced in ref. ^84^, is defined as

$${I}_{S,\mathrm{norm}}(T)=\frac{\mathop{\sum }\limits_{j=N-l+1}^{N}{\nu }_{j}-l}{\frac{1}{2}(l+2)(l-1)-l},$$

where to be able to compare indices of trees on different number of leaves *l*, we subtracted the minimal value for a given *l* and divided by the range of the index on all trees on *n* leaves, as in ref. ^85^.

For an interior node *i* of a binary tree *T*, we defined *T*_*L*_(*i*) as the number of leaves subtended by the left branch of *T*_*i*_, the subtree rooted at *i*, and *T*_*R*_(*i*) the number of leaves subtended by its right branch. Then, the unnormalized Colless index^86^ of *T* is

$${I}_{C}(T)=\mathop{\sum }\limits_{i=1}^{N-l}| {T}_{L}(i)-{T}_{R}(i)| .$$

Since Colless index is defined only for bifurcating trees, we used the default normalized Colless-like index ${{\mathfrak{C}}}_{{\mathrm{MDM}},\,{{\mathrm{ln}}}(l+e),\,{\mathrm{norm}}}$ defined in ref. ^85^. This consisted of measuring the dissimilarity between the subtrees $T^{\prime}$ rooted at a given internal node by computing the mean deviation from the median (MDM) of the *f*-sizes of these subtrees. In this case, $f(l)={{\mathrm{ln}}}(l+e)$ and the *f*-size of $T^{\prime}$ is defined as

$$\mathop{\sum}\limits_{v\in V(T^{\prime} )}{\mathrm{ln}}({\mbox{deg}}(v)+e).$$

These dissimilarities were then summed and the result was normalized as for Sackin’s index.

The cophenetic value *ϕ*(*i*, *j*) of a pair of leaves *i*, *j* is the depth of their lowest common ancestor (such that the root has depth 0). The total cophenetic index^87^ of *T* is then the sum of the cophenetic values over all pairs of leaves, and a normalized version is

$${I}_{{{\Phi }},{\mathrm{norm}}}(T)=\frac{\mathop{\sum}\limits_{N-l+1\le i < j\le N}\phi (i,j)}{\left({l}\atop{3}\right)},$$

where here the minimal value of the cophenetic index is 0 for all *l* (for a star-shaped tree with *l* leaves).

These three balance indices were designed for analysing species phylogenies and are thus defined on cladograms, which are trees in which leaves correspond to extant species and internal nodes are hypothetical common ancestors. Conventional cladograms have no notion of node size. Cladograms also lack linear components as each internal node necessarily corresponds to a branching event. The driver phylogenetic trees reported in multi-region sequencing studies and generated by our models are instead clone trees (also known as mutation trees), in which all nodes of non-zero size represent extant clones. To apply previous balance indices to driver phylogenetic trees, we first converted the trees to cladograms by adding a leaf to each non-zero-sized internal node and collapsing linear chains of zero-sized nodes.

Whereas diversity indices such as *D* are relatively robust to the addition or removal of rare clones, the balance indices described above are much less robust because they treat all clones equally, regardless of population size (Supplementary Figs. 6, 7 and 8). This hampered comparison between model results and data for two reasons. First, due to sampling error, even high quality multi-region sequencing studies underestimate the number of subclonal, locally abundant driver mutations by approximately 25%^81^. Second, bulk sequencing cannot detect driver mutations present in only a very small fraction of cells.

### A robust tree balance index

To overcome the shortcomings of previous indices, we have developed a more robust tree balance index based on an extended definition: tree balance is the degree to which internal nodes split the tree into subtrees of equal size, where size refers to the sum of all node populations.

Let *f*(*v*) > 0 denote the size of node *v*. For an internal node *i*, let *V*(*T*_*i*_) denote the set of nodes of *T*_*i*_, the subtree rooted at *i*. We then define

$$\begin{array}{l}{S}_{i}=\mathop{\sum}\limits_{v\in V({T}_{i})}f(v)=\,{{\mathrm{the}}\ {\mathrm{size}}\ {\mathrm{of}}}\,\,{T}_{i},\\ {S}_{i}^{* }=\mathop{\sum}\limits_{v\in V({T}_{i})\atop {v\ne i}}f(v)=\,{{\mathrm{the}}\ {\mathrm{size}}\ {\mathrm{of}}}\,\,{T}_{i}\,\,{{\mathrm{without}}\ {\mathrm{its}}\ {\mathrm{root}}}\,\,i.\end{array}$$

For *i* in the set of internal nodes $\widetilde{V}$, and *j* in the set *C*(*i*) of children of *i*, we define ${p}_{ij}={S}_{j}/{S}_{i}^{* }$. We then computed the balance score ${W}_{i}^{1}$ of a node $i\in \widetilde{V}$ as the normalized Shannon entropy of the sizes of the subtrees rooted at the children of *i*:

$${W}_{i}^{1}=\mathop{\sum}\limits_{j\in C(i)}{W}_{ij}^{1},\quad \,{{\mbox{with}}}\,{W}_{ij}^{1}=\left\{\begin{array}{ll}-{p}_{ij}{{{\mathrm{log}}}\,}_{{d}^{+}(i)}{p}_{ij}&\,{{\mbox{if}}}\,\,{p}_{ij} > 0\,{{\mbox{and}}}\,\,{d}^{+}(i)\ge 2,\\ 0&\,{{\mbox{otherwise,}}}\,\end{array}\right.$$

where *d*^+^(*i*) is the out-degree (the number of children) of node *i*. Finally, for each node *i*, we weighted the balance score by the product of ${S}_{i}^{* }$ and a non-root dominance factor ${S}_{i}^{* }/{S}_{i}.$ Our normalized balance index is then

$${J}^{1}:= \frac{1}{{\sum }_{k\in \widetilde{V}}{S}_{k}^{* }}\mathop{\sum}\limits_{i\in \widetilde{V}}{S}_{i}^{* }\frac{{S}_{i}^{* }}{{S}_{i}}{W}_{i}^{1}.$$

Supplementary Fig. 11 illustrates the calculation of *J*^1^ for four exemplary trees. We further describe the desirable properties of this index, and its relationship to other tree balance indices, in another article^43^.

When *n* ≤ 2 (where *n* is the mean number of driver mutations per cell), the non-root dominance factor cannot exceed *n* − 1, while the other factors in *J*^1^ are at most 1, which implies *J*^1^ ≤ *n* − 1 for all *n* ≤ 2. Also for *n* > 2, we have *J*^1^ ≤ 1 < *n* − 1. Thus, it is impossible to construct trees that have *J*^1^ > *n* − 1, as shown in Fig. 4a.

### Clonal turnover indices

For each time point *t* ≥ *δt*, we defined a clonal turnover index as

$${{\Theta }}(t)=\mathop{\sum}\limits_{i}{\left({f}_{i}(t)-{f}_{i}(t-\tau )\right)}^{2},$$

where *f*_*i*_(*t*) is the frequency of clone *i* at time *t*, and *τ* is 10% of the total simulation time measured in cell generations. The mean value $\overline{{{\Theta }}}$ over time measures the total extent of clonal turnover.

To determine whether clonal turnover mostly occurred early, late or throughout tumour evolution, we calculated the weighted average

$${\overline{T}}_{{{\Theta }}}=\frac{1}{\max (t)}\left(\mathop{\sum}\limits_{t}{{\Theta }}(t)t\bigg/\mathop{\sum}\limits_{t}{{\Theta }}(t)\right),$$

where $\max (t)$ denotes the final time of the simulation. This quantity takes values between 0 and 1, and is higher if clonal turnover occurs mostly late during tumour growth. If the rate of clonal turnover is constant over time, then ${\overline{T}}_{{{\Theta }}}\approx 0.55$.

### Histology slide analysis to determine the number of cells per gland

We randomly selected five tumours of each of four cancer types (colorectal cancer, clear cell renal cancer, lung adenocarcinoma and breast cancer) from The Cancer Genome Atlas (TCGA) reference database (http://portal.gdc.cancer.gov). Using QuPath v0.2.0m4^88^, we manually delineated five representative groups of tumour cells in each image and automatically counted the number of cells in each group. We defined a group as a set of tumour cells directly touching each other, separated from other groups by stroma or other non-tumour tissue (Extended Data Fig. 3).

The number of cells per group ranged from 5 to 8,485, with 50% of cases having between 53 and 387 cells (Extended Data Fig. 4a). Variation in the number of cells per group was larger between rather than within tumours, whereas cell density was relatively consistent between tumours (Extended Data Fig. 4b). Because our cell counts were derived from cross sections, they would underestimate the true populations of three-dimensional glands. On the other hand, it is unknown what proportion of cells are able to self-renew and contribute to long-term tumour growth and evolution^89^. On balance, therefore, it is reasonable to assume that each gland of an invasive glandular tumour can contain between a few hundred and a few thousand interacting cells. This range of values is, moreover, remarkably consistent with results of a recent study that used a very different method to infer the number of cells in tumour-originating niches. Across a range of tissue types, this study concluded that cells typically interact in communities of 300–1,900 cells^30^. Another recent study of breast cancer applied the Louvain method for community detection to identify two-dimensional tumour communities typically in the range of 10–100 cells.^29^

### Reporting Summary

Further information on research design is available in the Nature Research Reporting Summary linked to this article.

## Supplementary information

Supplementary Information The maximum possible diversity of linear trees, Supplementary Tables 1–4 and Figs. 1–11.

Reporting Summary

Peer Review Information

## Data Availability

Data can be accessed at https://github.com/robjohnnoble/ModesOfEvolution.

## Code availability

Our computational modelling code is available in an online repository^65^. Our data analysis R package is at https://github.com/robjohnnoble/demonanalysis. Specific figure plotting code is at https://github.com/robjohnnoble/ModesOfEvolution. Muller plots were drawn using the ggmuller R package^90^.
