# Supplementary material for: Spatial structure governs the mode of tumour evolution
Source: Nat Ecol Evol. 2021 Dec 23;6(2):207–17. doi: 10.1038/s41559-021-01615-9 (PMC8825284; doi:10.1038/s41559-021-01615-9)
Supplement: Supplementary file 1 — The maximum possible diversity of linear trees, Supplementary Tables 1–4 and Figs. 1–11. [file 41559_2021_1615_MOESM1_ESM.pdf]

nature portfolio

https://doi.org/10.1038/s41559-021-01615-9

Supplementary information

Spatial structure governs the mode of tumour evolution

In the format provided by the authors and unedited

SUPPLEMENTARY INFORMATION: SPATIAL STRUCTURE GOVERNS THE MODE OF TUMOUR EVOLUTION

The maximum possible diversity of linear trees

Here we derive for linear trees the maximum possible value of the inverse Simpson diversity index (D), as a function of the mean number n of driver mutations per cell.

Consider a linear tree of size N. For all $i = 1, 2, .., N$, let p_i denote the proportion of cells corresponding to node i, thus with i driver mutations. The mean number of driver mutations per cell is $n = \sum_{i=1}^{N} ip_i$. The inverse Simpson index is $D = 1/\sum_{i=1}^{N} p_i^2$. The maximum possible value of this index for a linear tree of size N is thus the value of the following maximization problem:

$$\max_{p \in K} 1 \Big/ \sum_{i=1}^{N} p_i^2 \tag{1}$$

where $K = \left\{ p = (p_1, ..., p_N) \in \mathbb{R}^N, p_i \geq 0 \text{ for all } i = 1, ..., N, \sum_{i=1}^{N} p_i = 1, \text{and} \sum_{i=1}^{N} ip_i = n \right\}$.

Proposition 1. *Assume $N \geq 3n - 1$. Let q denote the integer part of (i.e., the greatest integer no larger than) $3n - 1$. Let*

$$\mu_1 = \frac{4(3n - 1 - 2q)}{q(q - 1)}, \quad \mu_2 = \frac{12(q - 2n + 1)}{(q - 1)q(q + 1)}.$$

The solution of Problem (1) is unique and given by:

$$p_i^* = -\frac{1}{2}(\mu_1 + \mu_2 i) \text{ if } 1 \leq i \leq q, \text{ and } p_i^* = 0 \text{ otherwise.}$$

If $3n$ is an integer, then the value of this optimization problem is

$$D = \frac{(3n - 1)(3n - 2)}{4n - 2} = \frac{9(2n - 1)}{8} - \frac{1}{8(2n - 1)}.$$

Otherwise, the value of this optimization problem is:

$$D = \frac{(3n - 2 - \alpha)^2(3n - 1 - \alpha)(3n - \alpha)}{(3n - 2 - \alpha)(3n - \alpha)(4n - 2 - 2\alpha) + \alpha^2(4n - 2/3 - 4\alpha/3)},$$

where $\alpha = 3n - 1 - q$ is the fractional part of $3n - 1$, hence also of $3n$.

The value of Problem 1 is a nondecreasing function of tree size N (indeed, for any smaller linear tree, there is a linear tree of size N with the same value of D: just add artificial nodes with $p_i = 0$ at the end). Since by Proposition 1, the value of Problem (1) is the same for all $N \geq 3n - 1$, it follows that this is also the maximal value of the inverse Simpson index over all finite linear trees.

We now prove Proposition 1. For p in $\mathbb{R}^N$, let $f(p) = \sum_{i=1}^{N} p_i^2$, $h_1(p) = \sum_{i=1}^{N} p_i - 1$, $h_2(p) = \sum_{i=1}^{N} ip_i - n$, and let $g_i(p) = -p_i$ for all $i = 1, 2, ..., n$. Problem (1) is equivalent to the minimization problem:

$$\min_{p \in K} f(p), \text{ where } K = \{p \in \mathbb{R}^N, g_i(p) \leq 0, i = 1, ..., N, \text{ and } h_j(p) = 0, j = 1, 2\}. \tag{2}$$

Functions f and g_i, $i = 1, ..., N$, are at least weakly convex, and functions h_j, $j = 1, 2$, are affine. Problem (2) is thus a convex minimization problem. It follows that if $p \in K$ satisfies the well-known Karush-Kuhn-Tucker (KKT) conditions, then p is a solution of (2). The KKT conditions associated to this problem are: there exists real numbers μ_1, μ_2, λ_1, ..., λ_N, such that, for all $i = 1, ..., N$:

$$\begin{cases} 2p_i + \mu_1 + \mu_2 i - \lambda_i = 0 \\ \lambda_i \geq 0 \\ \lambda_i p_i = 0. \end{cases}$$

Assume $N \geq 3n - 1$. Let q be the largest integer no larger than $3n - 1$ (so $3n - 2 < q \leq 3n - 1 < q + 1$). Define μ_1, μ_2 and p^* as in Proposition 1. Finally, let $\lambda_i = 0$ if $1 \leq i \leq q$ and $\lambda_i = \mu_1 + \mu_2 i$ if $q + 1 \leq i \leq N$.

We first prove that $p^* \in K$: using the standard formulas

$$\sum_{i=1}^{q} i = q(q + 1)/2, \text{ and } \sum_{i=1}^{q} i^2 = q(q + 1)(2q + 1)/6,$$

it is easily seen that $\sum_{i=1}^{q} p_i^* = 1$ and $\sum_{i=1}^{q} i p_i^* = n$. We now check that $p_i^* \geq 0$ for all $i = 1, ..., n$. Since $p_i^* = 0$ for all $i \geq q+1$, it is enough to show that $p_i^* = 0$ for $i = 1, ..., q$. Since $q > 3n - 2 \geq 2n - 1 \geq 1$, it follows that $\mu_2 > 0$. Thus, p_i^* is decreasing for $i = 1, ..., q$, and $p_i^* \geq 0$ for all $i = 1, ..., q$ if $p_q^* \geq 0$. Computation shows that this is equivalent to $q \leq 3n - 1$, which holds by definition of q.

We now prove that p^* satisfies the KKT conditions. The first and third conditions are trivially satisfied by definition of p_i^* and λ_i. It remains to check that $\lambda_i \geq 0$ for all i. Since $\lambda_i = 0$ for $i \leq q$, it suffices to prove it for $i = q+1, ..., N$. But for $i \geq q+1$, $\lambda_i = \mu_1 + \mu_2 i$ is increasing in i, since $\mu_2 > 0$. Thus, it suffices to prove that $\lambda_{q+1} \geq 0$. Computation shows that this is equivalent to $q \geq 3n - 2$, which holds by definition of q.

It follows that p^* is solution of Problem (2), hence of Problem (1). The fact that this is the unique solution follows from the strict convexity of f and the convexity of K. The formula for the value D of Problem (1) then results from simple but tedious computation that we omit.

**Model**	**Selective sweeps**	**Progressive diversification**	**Branching**	**Effectively almost neutral**	**Above intermediate branching curve**
Non-spatial	99% (83%)	0% (0%)	1% (2%)	0% (15%)	1% (13%)
Gland fission	0% (4%)	98% (39%)	2% (23%)	0% (34%)	97% (83%)
Invasive glandular	4% (12%)	2% (25%)	94% (62%)	0% (1%)	56% (56%)
Boundary growth	1% (2%)	0% (0%)	0% (13%)	99% (85%)	78% (64%)

Supplementary table 1. Distribution of modes of tumour evolution observed in tumours simulated using different models. Four modes of tumour evolution are defined here in terms of n and D values, as in Table 1. The intermediate branching curve (final column) describes the maximum possible diversity of linear trees. The first percentage corresponds to the four non-neutral cohorts of simulations shown in Figure 3c (one set of parameter values per model). The second percentage (in parentheses) corresponds to the average of multiple cohorts with varied parameter values, as shown in Extended Data Figure 5 and Figures 1, 2 and 3.

**Study**	**Model type**	**Cells per deme**	**Within-deme selection?**	**Between-deme selection?**	**Maximum acquired drivers**	**Exterior**
Bozic *et al.* 2010[1]	Branching process	Not applicable	Not applicable	Not applicable	Unlimited	Void
Waclaw *et al.* 2015[2]	Eden model, voter model, or similar	1	No	Yes	Unlimited	Void
Sottoriva *et al.* 2015[3]	Deme fission	10,000	No	Yes	1	Void
Sun & Hu *et al.* 2017[4]	Deme fission (edge only)	1,000 or 10,000	Yes	No	1	Void
Current study	Any of the above	Any number	Yes	Yes	Unlimited	Tissue or void

Supplementary table 2. Comparison of selected models of tumour population genetics.

**Model**	**Gland size**	**Manner of cell dispersal**
Non-spatial	Effectively infinite	Not applicable
Gland fission	8,192	Glands bifurcate, such that each daughter gland inherits half of the original gland's population of cells
Invasive glandular	512	Individual cells disperse between neighbouring glands and invade normal tissue
Boundary growth	1	New cells are added to the edge of the tumour

Supplementary table 3. Characteristics of four example models.

Parameter	Value(s)
Deme carrying capacity, K	$1, 512, 2048, 8192, \infty$
Driver mutation rate per cell division	$10^{-6}, 10^{-5}, 10^{-4}$
Passenger mutation rate per cell division	0.1
Normal cell relative division rate	0.9
Cell death rate, relative to division rate, in non-spatial model	0.98
Mean value of driver effect on cell division rate	0, 0.05, 0.1, 0.2
Upper bound on cell division rate	10
Upper bound on dispersal rate	10
Dispersal rate	Conditional

SUPPLEMENTARY TABLE 4. Parameter values used in this study. Mutation rate is measured per cell division; division and dispersal rates are relative to the rates of the initial tumour cell. The effect of a driver mutation with effect size s is to multiply the trait value r by a factor of $1 + s(1 - r/m)$, where m is the upper bound. Dispersal rates are set such that tumours typically take between 500 and 1,000 cell generations to grow from one to one million cells, corresponding to several years of human tumour growth.

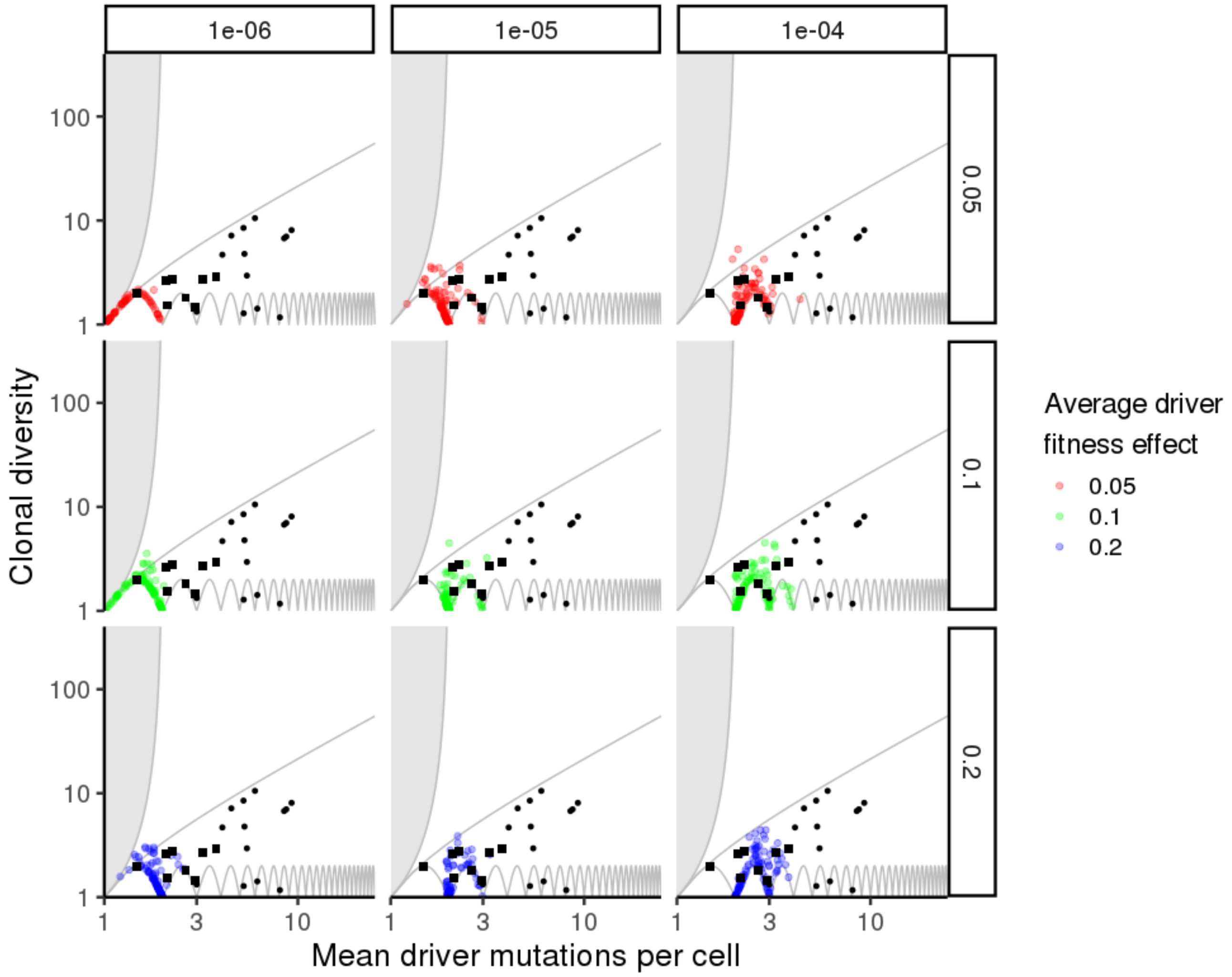

SUPPLEMENTARY FIGURE 1. Variation in evolutionary indices D and n for a non-spatial model. Results are shown for varied driver mutation rate (columns) and average driver fitness effect (rows), with 100 stochastic simulations per model. Large black squares show values derived from single-cell sequencing of acute myeloid leukaemia. Small black circles show values derived from multi-region sequencing of kidney cancers, lung cancers and breast cancers. Non-varied parameter values are the same as in Figure 2.

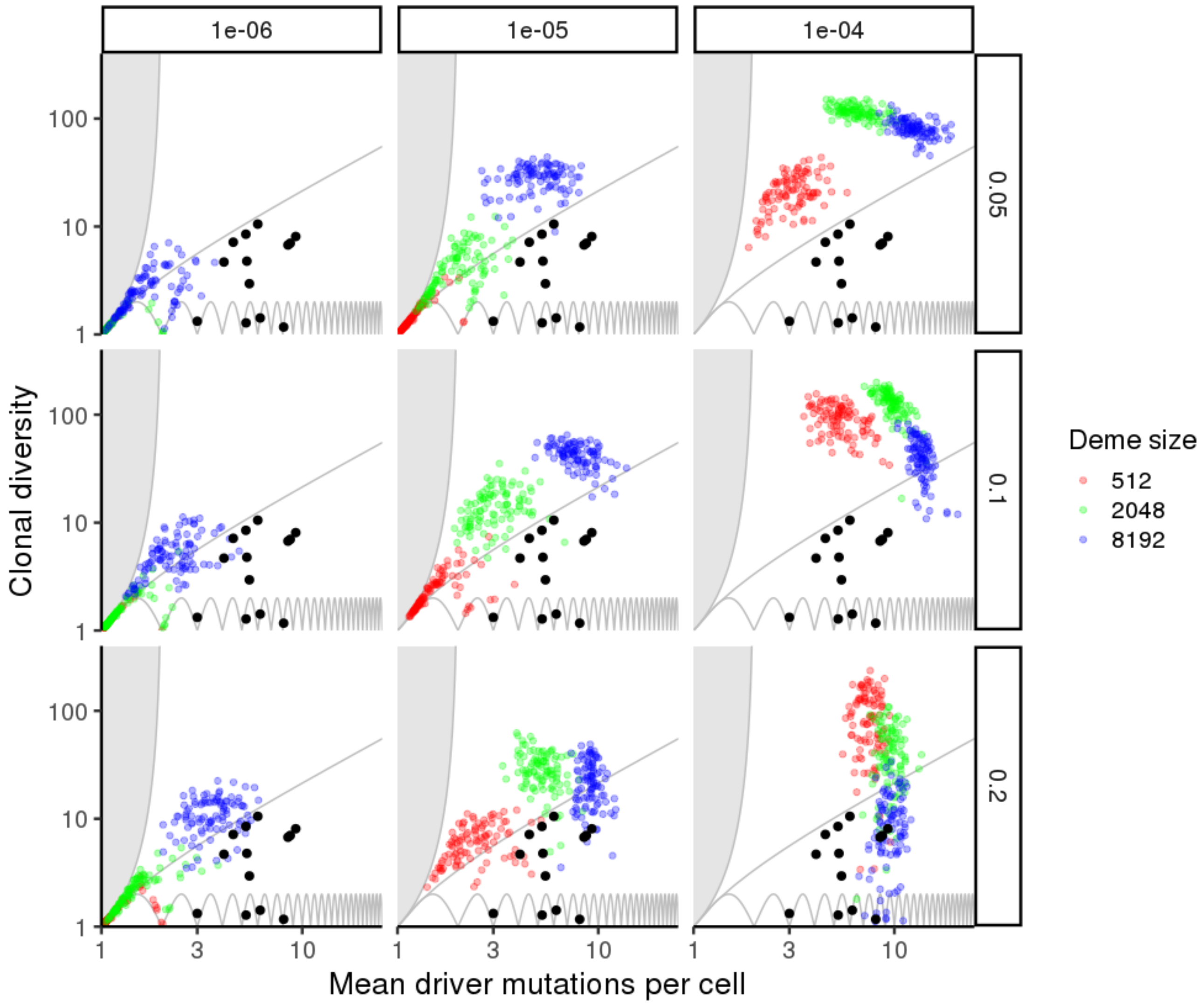

SUPPLEMENTARY FIGURE 2. Variation in evolutionary indices D and n for a gland fission model. Results are shown for varied gland size (colours), driver mutation rate (columns) and average driver fitness effect (rows), with 100 stochastic simulations per model. Black points show values derived from multi-region sequencing of kidney cancers, lung cancers and breast cancers. Non-varied parameter values are the same as in Figure 2.

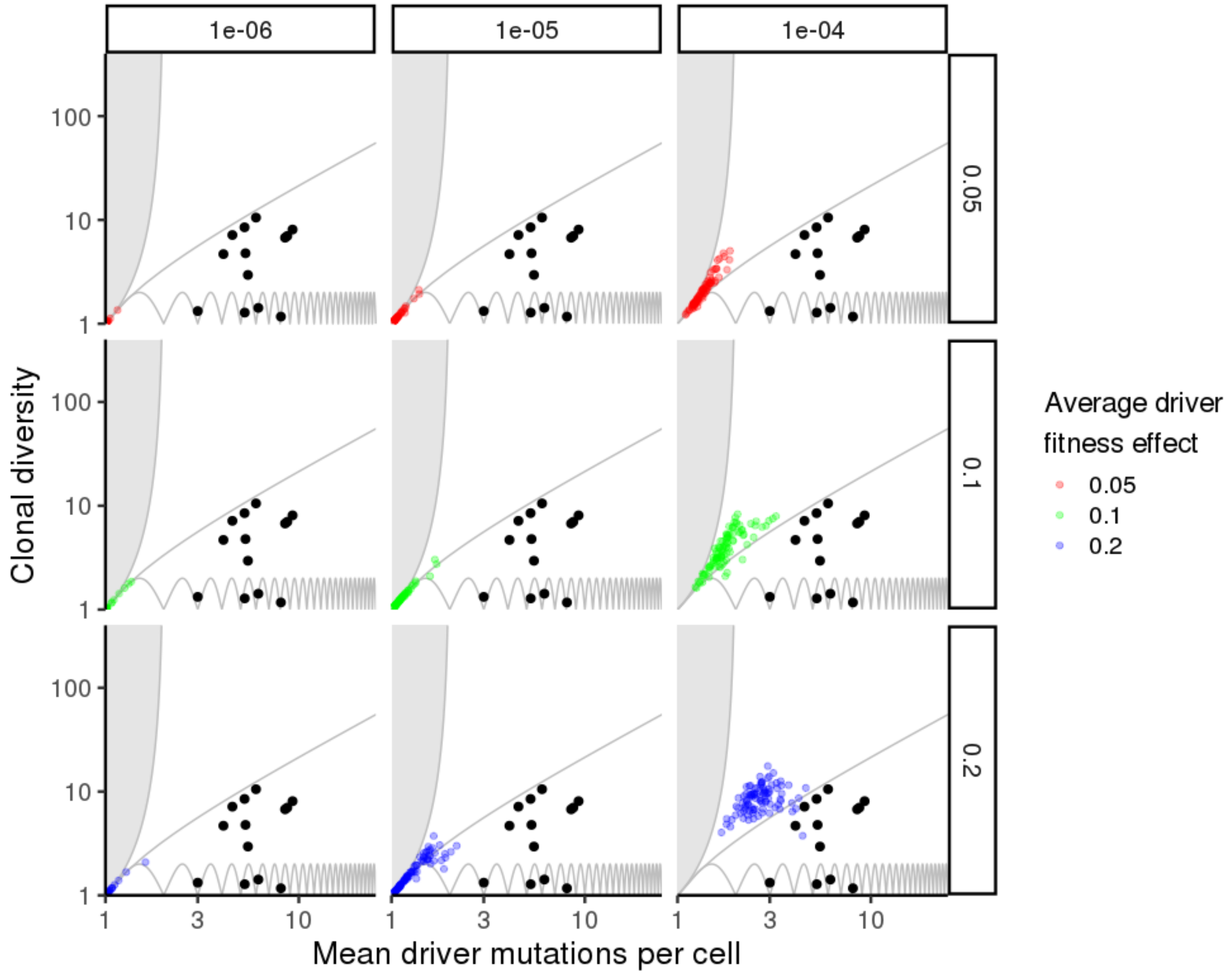

SUPPLEMENTARY FIGURE 3. Variation in evolutionary indices D and n for a boundary-growth model. Results are shown for varied driver mutation rate (columns) and average driver fitness effect (rows), with 100 stochastic simulations per model. Black points show values derived from multi-region sequencing of kidney cancers, lung cancers and breast cancers. Non-varied parameter values are the same as in Figure 2.

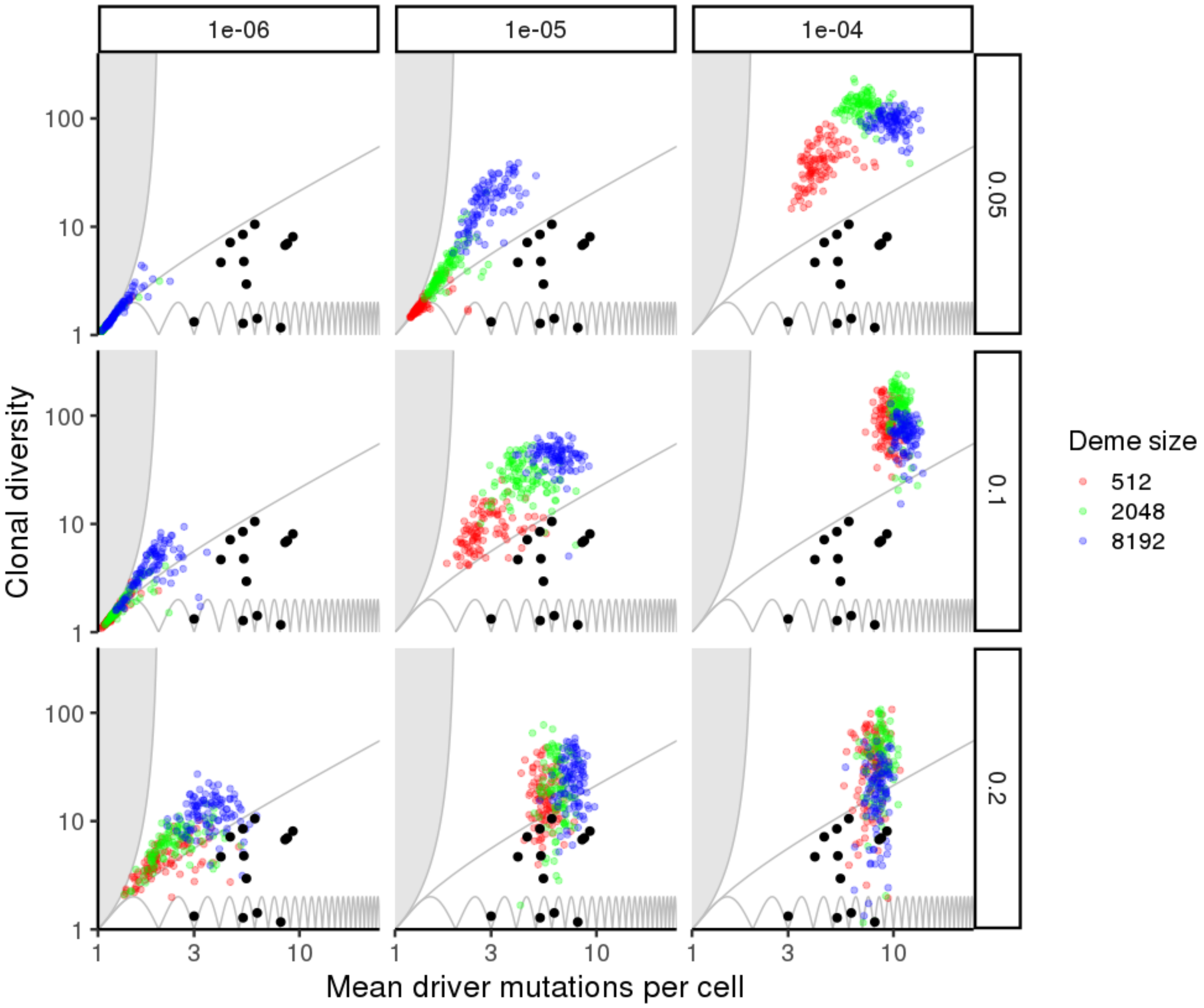

SUPPLEMENTARY FIGURE 4. Variation in evolutionary indices D and n for a glandular model without normal tissue. In this model, the space surrounding the tumour is assumed to be empty. Tumour cells disperse throughout the tumour as well as at the tumour boundary. Results are shown for varied gland size (colours), driver mutation rate (columns) and average driver fitness effect (rows), with 100 stochastic simulations per model. Black points show values derived from multi-region sequencing of kidney cancers, lung cancers and breast cancers. Non-varied parameter values are the same as in Figure 2.

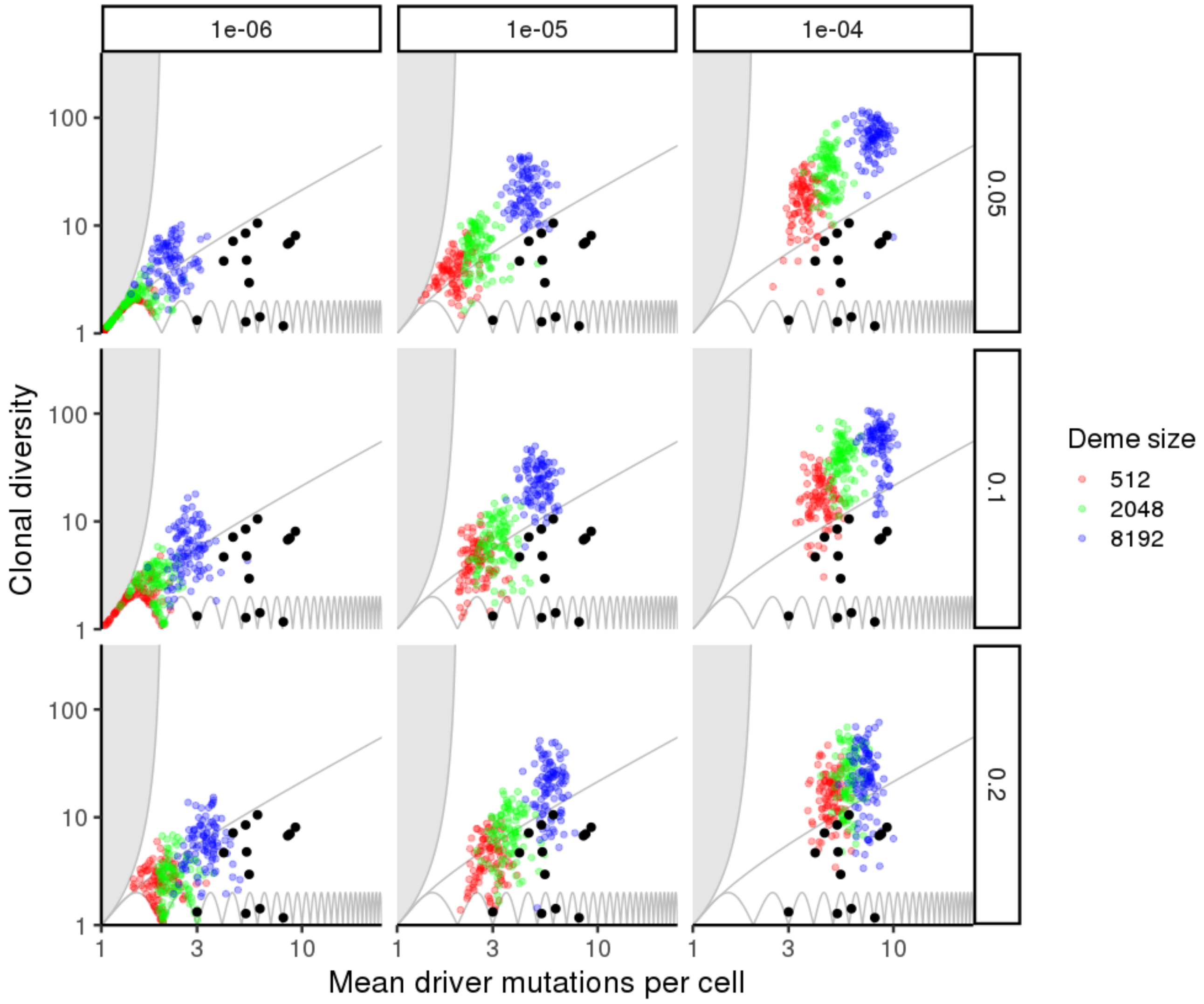

Supplementary figure 5. Variation in evolutionary indices D and n for an invasive glandular model with cell dispersal restricted to the tumour boundary. Results are shown for varied gland size (colours), driver mutation rate (columns) and average driver fitness effect (rows), with 100 stochastic simulations per model. Black points show values derived from multi-region sequencing of kidney cancers, lung cancers and breast cancers. Non-varied parameter values are the same as in Figure 2.

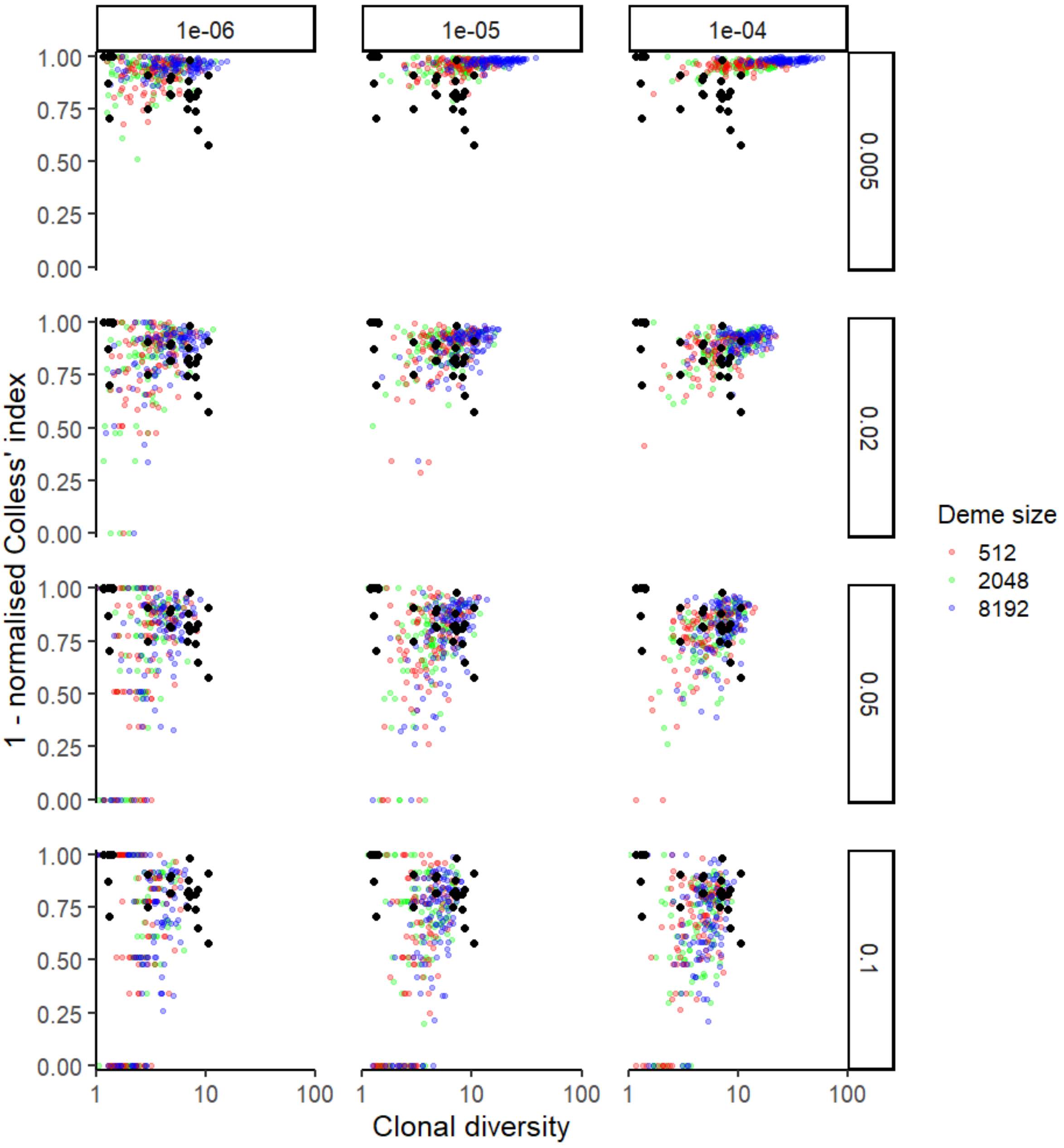

Supplementary figure 6. Variation in Colless's tree balance index versus clonal diversity D for an invasive glandular model with cell dispersal throughout the tumour and at the tumour boundary. Results are shown for varied gland size (colours), driver mutation rate (columns) and sensitivity threshold (rows), with 100 stochastic simulations per model. Driver mutations with frequency below the sensitivity threshold (0.005, 0.02, 0.05 or 0.1) are removed from the model output before calculating J^1 and D. Non-varied parameter values are the same as in Figure 2. Black points show values derived from multi-region sequencing of kidney cancers, lung cancers and breast cancers.

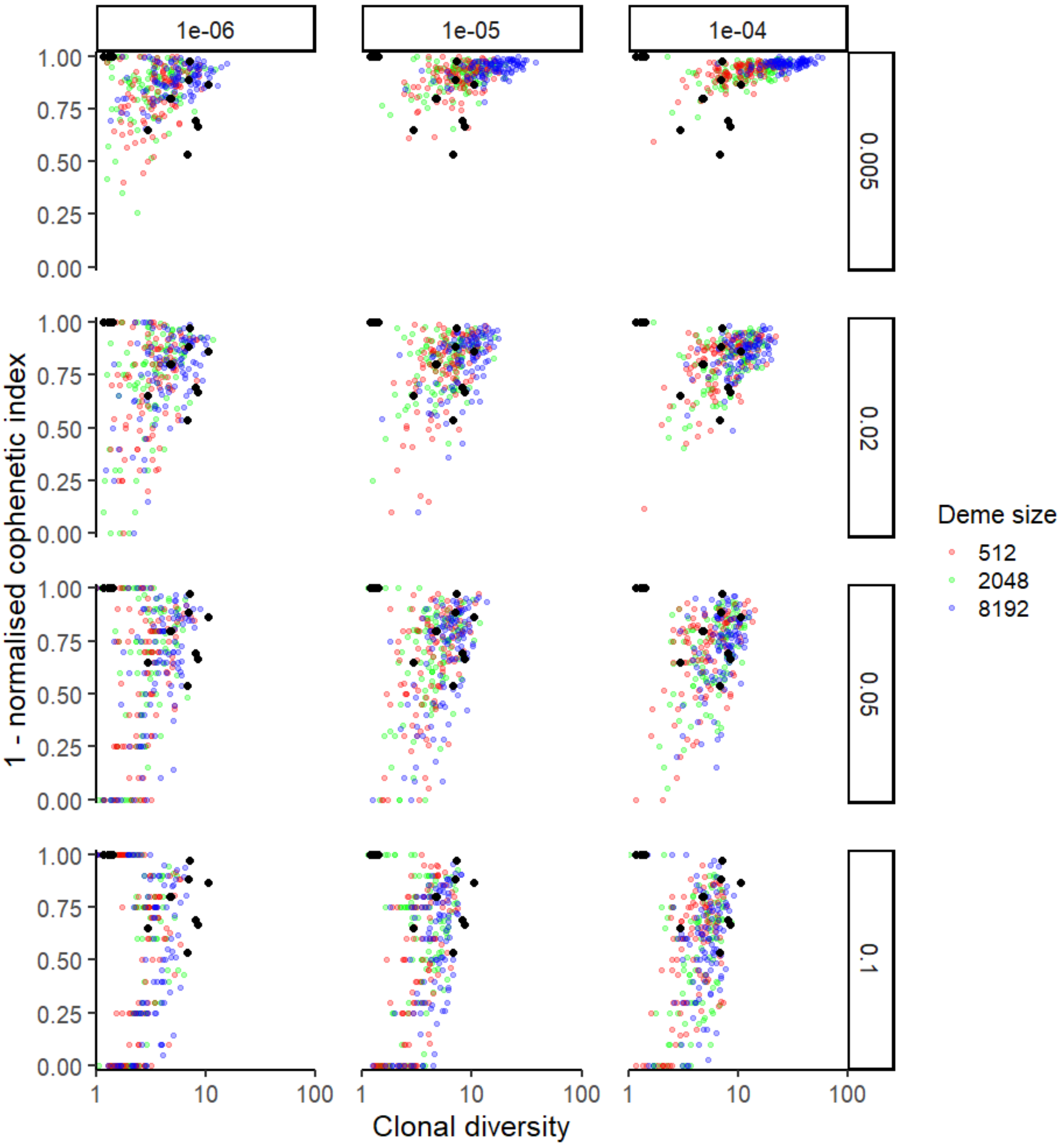

SUPPLEMENTARY FIGURE 7. Variation in the total cophenetic tree balance index versus clonal diversity D for an invasive glandular model with cell dispersal throughout the tumour and at the tumour boundary. Results are shown for varied gland size (colours), driver mutation rate (columns) and sensitivity threshold (rows), with 100 stochastic simulations per model. Driver mutations with frequency below the sensitivity threshold (0.005, 0.02, 0.05 or 0.1) are removed from the model output before calculating J^1 and D. Non-varied parameter values are the same as in Figure 2. Black points show values derived from multi-region sequencing of kidney cancers, lung cancers and breast cancers.

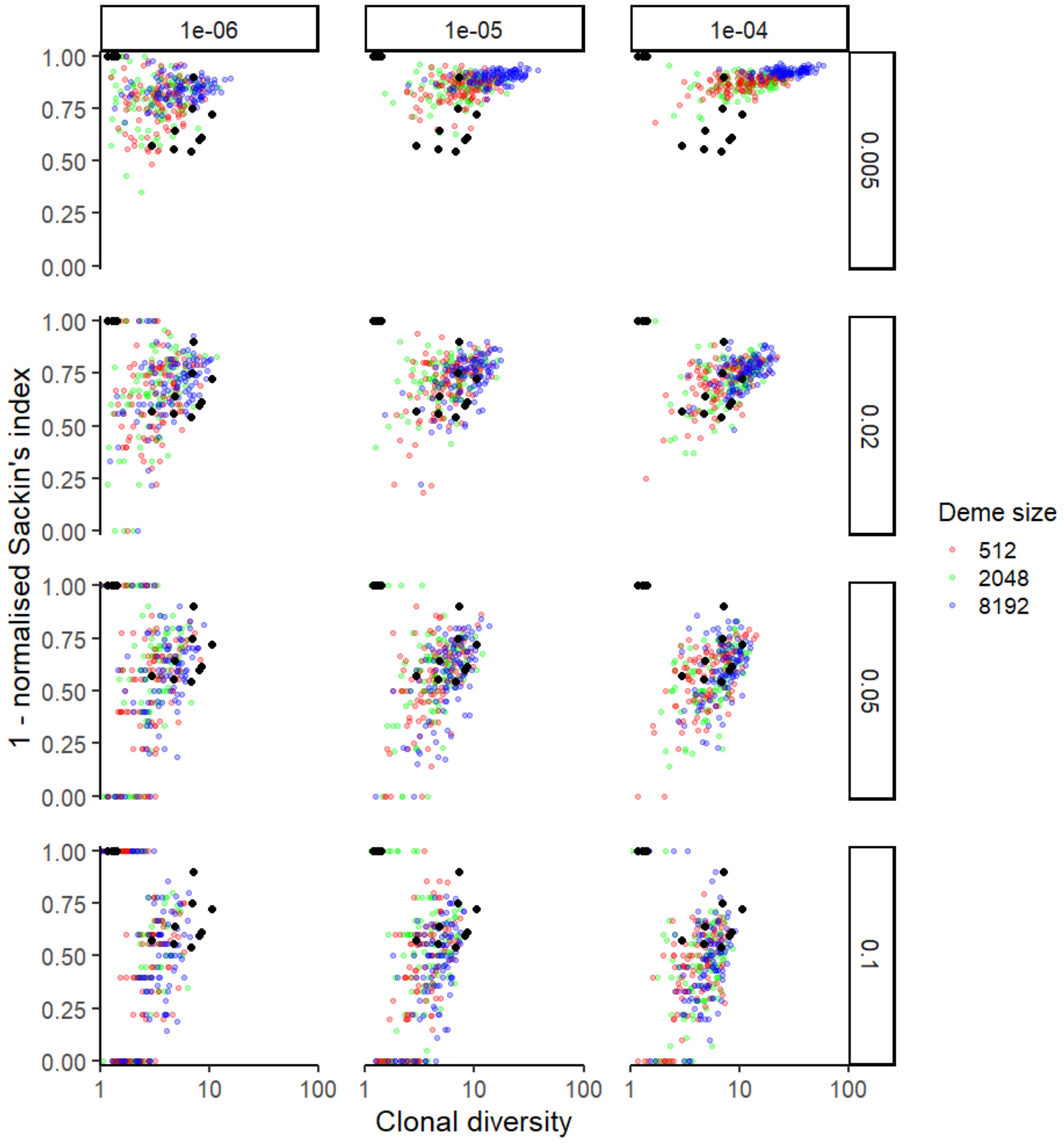

SUPPLEMENTARY FIGURE 8. Variation in Sackin's tree balance index versus clonal diversity D for an invasive glandular model with cell dispersal throughout the tumour and at the tumour boundary. Results are shown for varied gland size (colours), driver mutation rate (columns) and sensitivity threshold (rows), with 100 stochastic simulations per model. Driver mutations with frequency below the sensitivity threshold (0.005, 0.02, 0.05 or 0.1) are removed from the model output before calculating J^1 and D. Non-varied parameter values are the same as in Figure 2. Black points show values derived from multi-region sequencing of kidney cancers, lung cancers and breast cancers.

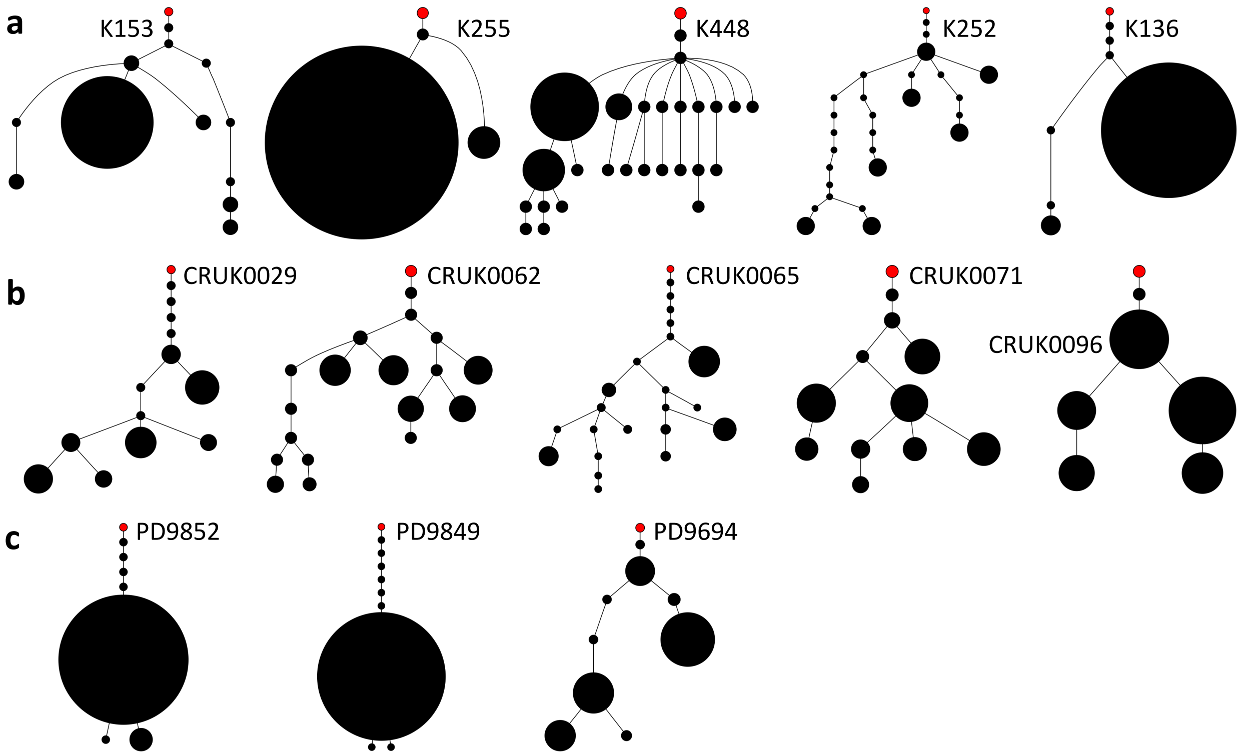

SUPPLEMENTARY FIGURE 9. Phylogenetic trees obtained from real tumours, without clustering driver mutations. Here we assume that all putative driver mutations were true drivers that occurred independently. **a**, Driver phylogenetic trees for five clear cell renal cell carcinomas, labelled with patient codes. Data was obtained from data set S2 of ref 5. Clone frequencies are estimated as the proportion of regions in which the corresponding combination of driver mutations was detected. **b**, Phylogenetic trees for five non-small-cell lung cancers, labelled with patient codes (from Figure S12 of ref 6). **c**, Phylogenetic trees for three breast cancers, labelled with patient codes (from Supplementary table S5 of ref 7). Node size corresponds to clone population size at the final time point and the founding clone is coloured red.

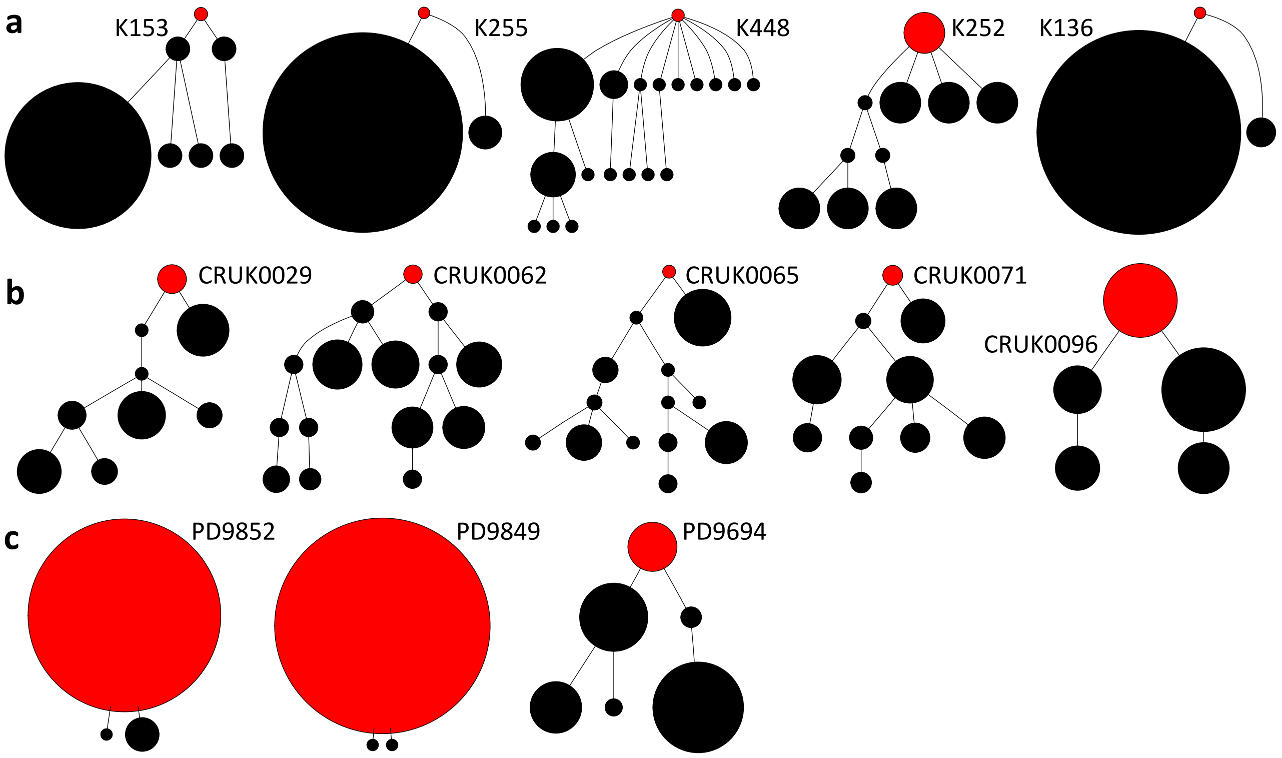

SUPPLEMENTARY FIGURE 10. Phylogenetic trees obtained from real tumours, after clustering driver mutations. Here we assume that each mutational cluster (a distinct peak in the variant allele frequency distribution) corresponds to exactly one driver mutation, while all other mutations are hitchhikers. **a**, Driver phylogenetic trees for five clear cell renal cell carcinomas, labelled with patient codes. Data was obtained from data set S2 of ref 5. Clone frequencies are estimated as the proportion of regions in which the corresponding combination of driver mutations was detected. **b**, Phylogenetic trees for five non-small-cell lung cancers, labelled with patient codes (from Figure S12 of ref 6). **c**, Phylogenetic trees for three breast cancers, labelled with patient codes (from Supplementary table S5 of ref 7). Node size corresponds to clone population size at the final time point and the founding clone is coloured red.

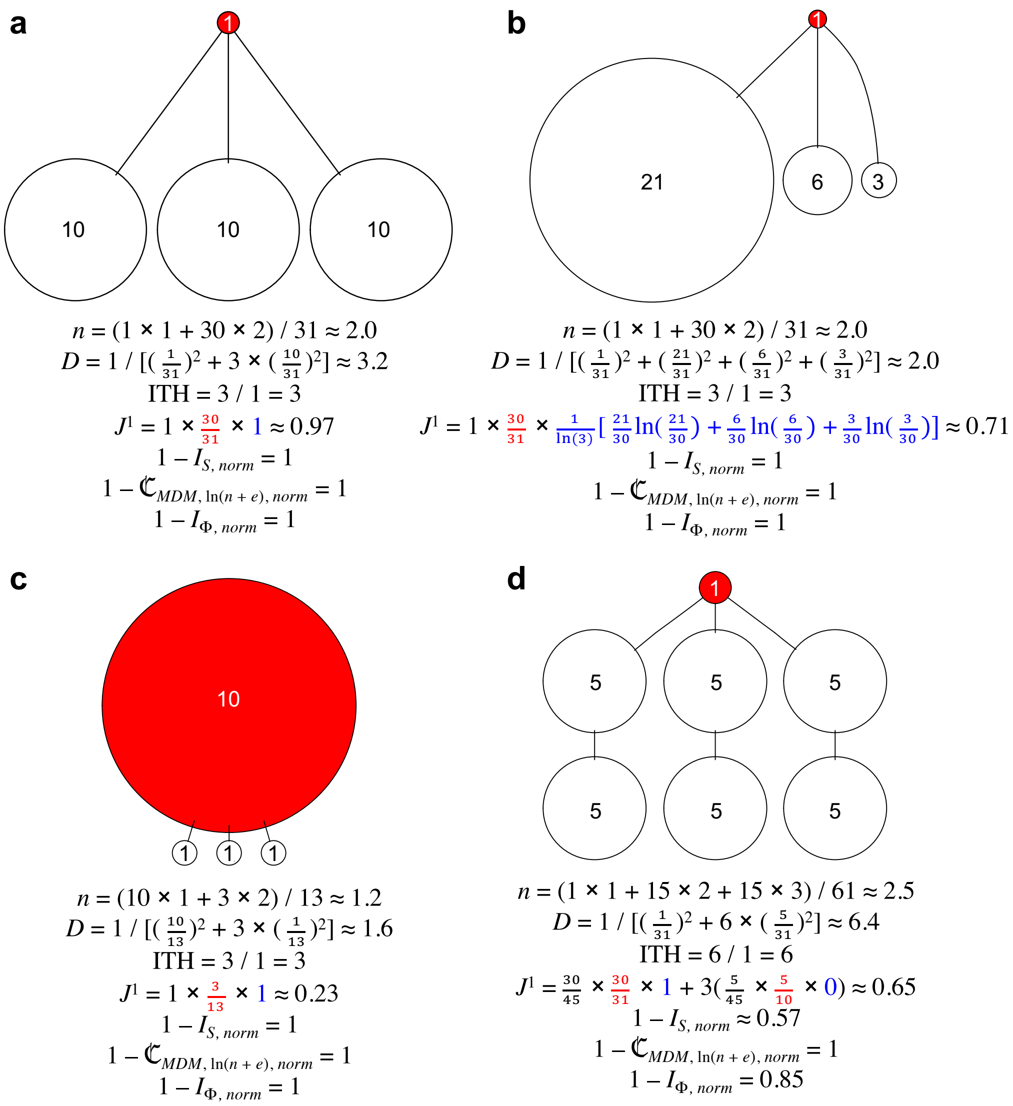

Supplementary figure 11. Summary indices for example tumour phylogenetic trees. Nodes are labelled with their relative sizes. The root is red. Index n is the mean number of driver mutations per cell; D is the inverse Simpson index; ITH is the ratio of subclonal to clonal driver mutations; J^1 is a general tree balance index; $I_{S,norm}$ is a normalised version of Sackin's tree balance index; $I_{\Phi,norm}$ is a normalised Colless-like tree balance index; $\mathfrak{C}_{MDM,\ln(n+e),norm}$ is a normalised version of the total cophenetic index. The ITH index and all tree balance indices except J^1 are identical for trees a, b and c because these indices ignore node sizes. Index D is lower for tree b than tree a, and lower for tree c than tree b, because D accounts for the degree of inequality among node sizes. Similarly, J^1 is lower for tree b than tree a because J^1 accounts for the degree of inequality among branch sizes (the third term in the product, in blue). J^1 is lower for tree c than tree a because the root node of tree c is more dominant (the second term in the product, in red). J^1 is lower for tree d than tree a because J^1 is a weighted average across all subtrees and tree d contains linear subtrees, which are considered unbalanced.

References

[1] Bozic, I. *et al.* Accumulation of driver and passenger mutations during tumor progression. *Proceedings of the National Academy of Sciences of the United States of America* **107**, 18545–50 (2010).

[2] Waclaw, B. *et al.* A spatial model predicts that dispersal and cell turnover limit intratumour heterogeneity. *Nature* **525**, 261–264 (2015).

[3] Sottoriva, A. *et al.* A Big Bang model of human colorectal tumor growth. *Nature Genetics* **47**, 209–216 (2015).

[4] Sun, R. *et al.* Between-region genetic divergence reflects the mode and tempo of tumor evolution. *Nature Genetics* **49**, 1015–1024 (2017).

[5] Turajlic, S. *et al.* Deterministic Evolutionary Trajectories Influence Primary Tumor Growth: TRACERx Renal. *Cell* **173**, 595–610.e11 (2018).

[6] Jamal-Hanjani, M. *et al.* Tracking the Evolution of Non–Small-Cell Lung Cancer. *New England Journal of Medicine* **376**, NEJMoa1616288 (2017).

[7] Yates, L. R. *et al.* Subclonal diversification of primary breast cancer revealed by multiregion sequencing. *Nature Medicine* **21** (2015).
