# Supplementary material for: Spatial structure governs the mode of tumour evolution
Source: Nat Ecol Evol. 2021 Dec 23;6(2):207–17. doi: 10.1038/s41559-021-01615-9 (PMC8825284; doi:10.1038/s41559-021-01615-9)
Supplement: Supplementary file 2 — Reporting Summary [file 41559_2021_1615_MOESM2_ESM.pdf]

nature portfolio

Corresponding author(s): Robert Noble

Last updated by author(s): Oct 6, 2021

Reporting Summary

Nature Portfolio wishes to improve the reproducibility of the work that we publish. This form provides structure for consistency and transparency in reporting. For further information on Nature Portfolio policies, see our Editorial Policies and the Editorial Policy Checklist.

Statistics

For all statistical analyses, confirm that the following items are present in the figure legend, table legend, main text, or Methods section.

n/a	Confirmed	
☐	☒	The exact sample size (*n*) for each experimental group/condition, given as a discrete number and unit of measurement
☐	☒	A statement on whether measurements were taken from distinct samples or whether the same sample was measured repeatedly
☒	☐	The statistical test(s) used AND whether they are one- or two-sided<br>*Only common tests should be described solely by name; describe more complex techniques in the Methods section.*
☐	☒	A description of all covariates tested
☒	☐	A description of any assumptions or corrections, such as tests of normality and adjustment for multiple comparisons
☐	☒	A full description of the statistical parameters including central tendency (e.g. means) or other basic estimates (e.g. regression coefficient) AND variation (e.g. standard deviation) or associated estimates of uncertainty (e.g. confidence intervals)
☒	☐	For null hypothesis testing, the test statistic (e.g. *F*, *t*, *r*) with confidence intervals, effect sizes, degrees of freedom and *P* value noted<br>*Give P values as exact values whenever suitable.*
☒	☐	For Bayesian analysis, information on the choice of priors and Markov chain Monte Carlo settings
☒	☐	For hierarchical and complex designs, identification of the appropriate level for tests and full reporting of outcomes
☒	☐	Estimates of effect sizes (e.g. Cohen's *d*, Pearson's *r*), indicating how they were calculated

Our web collection on statistics for biologists contains articles on many of the points above.

Software and code

Policy information about availability of computer code

Data collection	Our computational modelling code used to generate data is available at https://github.com/robjohnnoble/demon model.
Data analysis	Our data analysis R package is at https://github.com/robjohnnoble/demon_analysis. Specific figure plotting code is at https://github.com/robjohnnoble/ModesOfEvolution. We used QuPath for image analysis (https://qupath.github.io). We also used the R packages ggmuller (https://cran.r-project.org/package=ggmuller), cluster (https://cran.r-project.org/web/packages/cluster/index.html), and CollessLike (https://github.com/cran/CollessLike).

For manuscripts utilizing custom algorithms or software that are central to the research but not yet described in published literature, software must be made available to editors and reviewers. We strongly encourage code deposition in a community repository (e.g. GitHub). See the Nature Portfolio guidelines for submitting code & software for further information.

Data

Policy information about availability of data

All manuscripts must include a data availability statement. This statement should provide the following information, where applicable:

- Accession codes, unique identifiers, or web links for publicly available datasets
- A description of any restrictions on data availability
- For clinical datasets or third party data, please ensure that the statement adheres to our policy

Data can be accessed at https://github.com/robjohnnoble/ModesOfEvolution.

Field-specific reporting

Please select the one below that is the best fit for your research. If you are not sure, read the appropriate sections before making your selection.

☒ Life sciences ☐ Behavioural & social sciences ☐ Ecological, evolutionary & environmental sciences

For a reference copy of the document with all sections, see nature.com/documents/nr-reporting-summary-flat.pdf

Life sciences study design

All studies must disclose on these points even when the disclosure is negative.

Sample size	For our image analysis we used 100 tumour glands from twenty tumours representing four cancer types, which was more than sufficient to estimate the mean number of cells per tumour gland within an order of magnitude, as required for parameterising our models. In our modelling we used 100 stochastic replicates per parameterisation, which was sufficient to represent distributions of outcomes.
Data exclusions	From each of the seven cohorts we obtained data for between three and eight tumours. In the ccRCC data set, we focussed on the five tumours for which driver frequencies were reported in the original publication. For NSCLC, we used data for the five tumours for which at least six multi-region samples were sequenced. In mesothelioma, we selected the six tumours that had at least five samples taken. From the breast cancer multi-region study, we used data for the three untreated tumours that were subjected to multi-region sequencing. From the single-cell sequencing studies of uveal melanoma and breast cancer we used all the published data (eight tumours in each case), and from the AML study we selected a random sample of eight tumours.
Replication	We report no experimental findings.
Randomization	We report no experimental findings.
Blinding	We report no experimental findings.

Reporting for specific materials, systems and methods

We require information from authors about some types of materials, experimental systems and methods used in many studies. Here, indicate whether each material, system or method listed is relevant to your study. If you are not sure if a list item applies to your research, read the appropriate section before selecting a response.

Materials & experimental systems

n/a	Involved in the study	
☒	☐	Antibodies
☒	☐	Eukaryotic cell lines
☒	☐	Palaeontology and archaeology
☒	☐	Animals and other organisms
☒	☐	Human research participants
☒	☐	Clinical data
☒	☐	Dual use research of concern

Methods

n/a	Involved in the study	
☒	☐	ChIP-seq
☒	☐	Flow cytometry
☒	☐	MRI-based neuroimaging
