# Supplementary material for: Spatial structure governs the mode of tumour evolution
Source: Nat Ecol Evol. 2021 Dec 23;6(2):207–17. doi: 10.1038/s41559-021-01615-9 (PMC8825284; doi:10.1038/s41559-021-01615-9)
Supplement: Supplementary file 3 — Peer Review Information [file 41559_2021_1615_MOESM3_ESM.pdf]

natureresearch

Peer Review Information

Journal: Nature Ecology & Evolution
Manuscript Title: Spatial structure governs the mode of tumour evolution
Corresponding author name(s): Robert Noble, Niko Beerenwinkel

Editorial Notes:

**Transferred manuscripts**	This manuscript has been previously reviewed at another journal that is not operating a transparent peer review scheme. This document only contains reviewer comments, rebuttal and decision letters for versions considered at Nature XX.
**Transferred manuscripts (no peer review at Nature XX)**	This manuscript has been previously reviewed at another journal that is not operating a transparent peer review scheme. The manuscript was considered suitable for publication without further review at Nature XX.
**Redactions – transferred manuscripts (mention of previous referee reports from elsewhere)**	This manuscript has been previously reviewed at another journal. This document only contains reviewer comments, rebuttal and decision letters for versions considered at Nature XX. Mentions of prior referee reports have been redacted
**Redactions – transferred manuscripts (mention of the other journal)**	This manuscript has been previously reviewed at another journal. This document only contains reviewer comments, rebuttal and decision letters for versions considered at Nature XX. Mentions of the other journal have been redacted.
**Redactions – unpublished data**	Parts of this Peer Review File have been redacted as indicated to maintain the confidentiality of unpublished data.
**Redactions – confidential patient information**	Parts of this Peer Review File have been redacted as indicated to maintain patient confidentiality.
**Redactions – published data**	Parts of this Peer Review File have been redacted as indicated to remove third-party material.
**Redactions – reviewer opt-out**	Parts of this Peer Review File have been redacted as indicated as we could not obtain permission to publish the reports of reviewer no. XX.
**Reviewer comments in marked-up manuscript**	In their review of the [first/second/third/...] version of this manuscript, reviewer no. XX added their comments to the manuscript file. These comments, excluding minor textual revisions, have been copied into this Peer Review File.

Reviewer Comments & Decisions:

Decision Letter, initial version:

8th September 2021

Dear Dr. Beerenwinkel,

Thank you for submitting your revised manuscript "Spatial structure governs the mode of tumour evolution" (NATECOLEVOL-210613928-T). I'm writing in place of my colleague Patrick Goymer, who is now on leave until the end of this year.

We have now received comments on the revised paper from the reviewer to whom we sent it - this person is our Reviewer 1 but they were Reviewer 2 from the previous round of reviews at Nature Genetics. I'm sorry for the confusion on numbering and thank you for your patience while we gathered this review.

As you will see from the comments pasted below, the reviewer finds that the paper has improved in revision, and therefore we'll be happy in principle to publish it in Nature Ecology & Evolution, pending minor revisions to satisfy the reviewers' final requests and to comply with our editorial and formatting guidelines.

At this point, we would also like you to re-visit the comments from the previous round of review at Nature Genetics, as there were some comments from the Reviewer 1 of that round to which we have not seen a point-by-point response, and we would like to verify that those comments were addressed in the revision we received (or to hear from you why there were no changes made).

If the current version of your manuscript is in a PDF format, please email us a copy of the file in an editable format (Microsoft Word or LaTex)-- we can not proceed with PDFs at this stage.

We are now performing detailed checks on your paper and will send you a checklist detailing our editorial and formatting requirements in about a week. Please do not upload the final materials and make any revisions until you receive this additional information from us.

Thank you again for your interest in Nature Ecology & Evolution. Please do not hesitate to contact me if you have any questions.

[REDACTED]

Reviewer #1 (Remarks to the Author):

The authors have extensively revised and updated the manuscript since I first reviewed it. They now strengthened the justification for the selection of growth patterns and their model parametrisation. They show that their results are in line with observations in several multi-region and single cell sequencing studies in solid tumors and leukemias, suggesting that these models provides a

meaningful representation of tumor evolution. I only have one further comment which the authors should address to avoid misunderstanding. The manuscript appears otherwise suitable for publication.

Comment:
The term gene flow is used several times in the paper. To my knowledge, it is specifically used in the context of sexually reproducing organisms or horizontal gene transfer in evolutionary biology. This may need to be replaced by a more suitable term for the strictly asexual model of cancer evolution that has been used by the authors.

Our ref: NATECOLEVOL-210613928-T

9th September 2021

Dear Dr. Beerenwinkel,

Thank you for your patience as we've prepared the guidelines for final submission of your Nature Ecology & Evolution manuscript, "Spatial structure governs the mode of tumour evolution" (NATECOLEVOL-210613928-T). Please carefully follow the step-by-step instructions provided in the attached file, and add a response in each row of the table to indicate the changes that you have made. Ensuring that each point is addressed will help to ensure that your revised manuscript can be swiftly handed over to our production team.

We would like to start working on your revised paper, with all of the requested files and forms, as soon as possible (preferably within two weeks). Please get in contact with us immediately if you anticipate it taking more than two weeks to submit these revised files.

When you upload your final materials, please include a point-by-point response to any remaining reviewer comments.

If you have not done so already, please alert us to any related manuscripts from your group that are under consideration or in press at other journals, or are being written up for submission to other journals (see: https://www.nature.com/nature-research/editorial-policies/plagiarism#policy-on-duplicate-publication for details).

In recognition of the time and expertise our reviewers provide to Nature Ecology & Evolution's editorial process, we would like to formally acknowledge their contribution to the external peer review of your manuscript entitled "Spatial structure governs the mode of tumour evolution". For those reviewers who give their assent, we will be publishing their names alongside the published article.

Nature Ecology & Evolution offers a Transparent Peer Review option for new original research manuscripts submitted after December 1st, 2019. As part of this initiative, we encourage our authors to support increased transparency into the peer review process by agreeing to have the reviewer comments, author rebuttal letters, and editorial decision letters published as a Supplementary item. When you submit your final files please clearly state in your cover letter whether or not you would like

to participate in this initiative. Please note that failure to state your preference will result in delays in accepting your manuscript for publication.

Cover suggestions

As you prepare your final files we encourage you to consider whether you have any images or illustrations that may be appropriate for use on the cover of Nature Ecology & Evolution.

Covers should be both aesthetically appealing and scientifically relevant, and should be supplied at the best quality available. Due to the prominence of these images, we do not generally select images featuring faces, children, text, graphs, schematic drawings, or collages on our covers.

We accept TIFF, JPEG, PNG or PSD file formats (a layered PSD file would be ideal), and the image should be at least 300ppi resolution (preferably 600-1200 ppi), in CMYK colour mode.

If your image is selected, we may also use it on the journal website as a banner image, and may need to make artistic alterations to fit our journal style.

Please submit your suggestions, clearly labeled, along with your final files. We'll be in touch if more information is needed.

Nature Ecology & Evolution has now transitioned to a unified Rights Collection system which will allow our Author Services team to quickly and easily collect the rights and permissions required to publish your work. Approximately 10 days after your paper is formally accepted, you will receive an email in providing you with a link to complete the grant of rights. If your paper is eligible for Open Access, our Author Services team will also be in touch regarding any additional information that may be required to arrange payment for your article.

Please note that <i>Nature Ecology & Evolution</i> is a Transformative Journal (TJ). Authors may publish their research with us through the traditional subscription access route or make their paper immediately open access through payment of an article-processing charge (APC). Authors will not be required to make a final decision about access to their article until it has been accepted. Find out more about Transformative Journals

<B>Authors may need to take specific actions to achieve compliance with funder and institutional open access mandates. For submissions from January 2021, if your research is supported by a funder that requires immediate open access (e.g. according to Plan S principles) then you should select the gold OA route, and we will direct you to the compliant route where possible. For authors selecting the subscription publication route our standard licensing terms will need to be accepted, including our self-archiving policies. Those standard licensing terms will supersede any other terms that the author or any third party may assert apply to any version of the manuscript.

Please note that you will not receive your proofs until the publishing agreement has been received through our system.

For information regarding our different publishing models please see our Transformative Journals page. If you have any questions about costs, Open Access requirements, or our legal forms, please contact ASJournals@springernature.com.

Please use the following link for uploading these materials:
[REDACTED]

If you have any further questions, please feel free to contact me.

[REDACTED]

Reviewer #1:
Remarks to the Author:
The authors have extensively revised and updated the manuscript since I first reviewed it. They now strengthened the justification for the selection of growth patterns and their model parametrisation. They show that their results are in line with observations in several multi-region and single cell sequencing studies in solid tumors and leukemias, suggesting that these models provides a meaningful representation of tumor evolution. I only have one further comment which the authors should address to avoid misunderstanding. The manuscript appears otherwise suitable for publication.

Comment:
The term gene flow is used several times in the paper. To my knowledge, it is specifically used in the context of sexually reproducing organisms or horizontal gene transfer in evolutionary biology. This may need to be replaced by a more suitable term for the strictly asexual model of cancer evolution that has been used by the authors.

Author Rebuttal to Initial comments

As requested, what follows is a point-by-point response to the remaining reviewer comments from NEE Reviewer 1, and the comments of Reviewer 1 from the last round of review at Nature Genetics.

NEE Reviewer 1 remaining comments:

The term gene flow is used several times in the paper. To my knowledge, it is specifically used in the context of sexually reproducing organisms or horizontal gene transfer in evolutionary biology. This may need to be replaced by a more suitable term for the strictly asexual model of cancer evolution that has been used by the authors.

As we wrote in our response to the first round of reviews (for Nature), after reviewing literature and consulting colleagues with relevant expertise, we have been unable to find any authoritative reference supporting the reviewer's claim. A typical conventional definition is that "Gene flow is a collective term that includes all mechanisms resulting in the movement of genes from one population to another" [Slarkin, M. Annual Review of Ecology and Systematics 16.1 (1985): 393-430]. We now cite Slarkin's paper to support our use of this term in the context of asexual tumour evolution.

Nature Genetics Reviewer #1 Remarks to the Author:

I reviewed this manuscript before, and was very positive about it then, and remain so now. I asked for a number of difficult/laborious revisions, the vast majority of which were taken under advisement and performed -- yielding a much stronger manuscript. I recommend accepting this manuscript as is, and have only a small number of queries which could be safely left for future work/consideration (except a couple typos which might as well be fixed).

We agree with the reviewer that these extensive revisions have strengthened our manuscript.

typos/clarifications: line 64: simulation -> simulating

We have corrected this typo.

line 108: can the authors define 'relatively large nodes' here?

Previously we wrote that clonal diversity "roughly corresponds to the number of relatively large nodes in the driver phylogenetic tree". We acknowledge that this was somewhat unclear. We have rephrased to say that the clonal diversity index "grows with the number of large nodes". We think this provides sufficient intuition given that the Simpson diversity index is well known to evolutionary biologists and ecologists and has been extensively described in numerous papers.

Fig 2k and many of the supplemental figures seem to be low resolution

All main figures, extended data figures, and supplementary figures are now provided either in high resolution or as vector graphics.

Figure 2 (and in general) can the authors please give a reason for the chosen number of cells/gland etc.

In the Methods (subsection “Histology slide analysis to determine the number of cells per gland”) we now cite additional studies to support our parametrization.

I'm a little confused by Figure 3: it seems to me that the colorbars show that where there is a high number of passenger mutations, there is reduced growth rate. while passenger mutations do not carry any change to growth rate themselves (they are neutral, right?), they should be a surrogate for driver mutations which occur at 1e-5, but confer a strictly positive growth rate advantage. so, two questions: why choose to show us an enumeration of passengers instead of drivers (or both would have worked)? and why am i seeing this anti-correlation? what am I missing? Please let me know here if i'm just missing something, or clarify in the text if it has been left out.

In the former Figure 3, which is now Extended Data Figure 1, we show both the mean cell proliferation rate and the number of passengers per cell. We do not also show the number of drivers per cell because this would not provide much extra information, given that each additional driver increases the proliferation rate. Passenger mutations are indeed neutral in our model. Whereas the reviewer refers to an anti-correlation, the first two rows of Extended Data Figure 1 in fact show positive correlations between the number of passengers per cell and the mean cell proliferation rate (pink regions in panels a and c tend to correspond to pink regions in panels b and d), consistent with the reviewer’s expectation. We now mention this positive correlation in the main text. The third row of this figure shows no clear pattern because there is insufficient variation in mean cell proliferation rate. So, we are unsure what anti-correlation the reviewer is referring to; perhaps the reviewer simply misread the colour scales.

ok: now three things I'm curious about for either future work or just to think about.

The remaining comments, though helpful and interesting, do not pertain to suggested changes. We now cite the mentioned paper by West et al. (Nat Comms 2021) in our Discussion.

natureresearch

Final Decision Letter:
